# Chemical evolution of ASO-like DNAzymes for effective and extended gene silencing in cells

**DOI:** 10.1093/nar/gkaf144

**Published:** 2025-03-04

**Authors:** Yingyu Liu, Sheyu Zhang, Meiqi Zhang, Xin Liu, Yashu Wu, Qin Wu, John C Chaput, Yajun Wang

**Affiliations:** Hangzhou Institute of Medicine (HIM), Chinese Academy of Sciences, Hangzhou, Zhejiang 310000, China; Hangzhou Institute of Medicine (HIM), Chinese Academy of Sciences, Hangzhou, Zhejiang 310000, China; Hangzhou Institute of Medicine (HIM), Chinese Academy of Sciences, Hangzhou, Zhejiang 310000, China; Hangzhou Institute for Advanced Study, University of Chinese Academy of Sciences, Hangzhou, Zhejiang 310024, China; Hangzhou Institute of Medicine (HIM), Chinese Academy of Sciences, Hangzhou, Zhejiang 310000, China; College of Pharmaceutical Science, Zhejiang University of Technology, Hangzhou, Zhejiang 310014, China; Hangzhou Institute of Medicine (HIM), Chinese Academy of Sciences, Hangzhou, Zhejiang 310000, China; Hangzhou Institute of Medicine (HIM), Chinese Academy of Sciences, Hangzhou, Zhejiang 310000, China; Zhejiang Cancer Hospital, The University of Chinese Academy of Sciences, Hangzhou, Zhejiang 310022, China; Department of Pharmaceutical Sciences, University of California, Irvine, CA 92697-3958, United States; Department of Chemistry, University of California, Irvine, CA 92697-3958, United States; Department of Molecular Biology and Biochemistry, University of California, Irvine, CA 92697-3958, United States; Department of Chemical and Biomolecular Engineering, University of California, Irvine, CA 92697-3958, United States; Hangzhou Institute of Medicine (HIM), Chinese Academy of Sciences, Hangzhou, Zhejiang 310000, China; Zhejiang Cancer Hospital, The University of Chinese Academy of Sciences, Hangzhou, Zhejiang 310022, China

## Abstract

Antisense oligonucleotides (ASOs) and small interfering RNA (siRNA) therapeutics highlight the power of oligonucleotides in silencing disease-causing messenger RNAs (mRNAs). Another promising class of gene-silencing oligonucleotides is RNA-cleaving nucleic acid enzymes, which offer the potential for allele-specific RNA inhibition with greater precision than ASOs and siRNAs. Herein, we chemically evolved the nucleolytic DNA enzyme (DNAzyme) 10–23, by incorporating the modifications that are essential to the success of ASO drugs, including 2′-fluoro, 2′-O-methyl, and 2′-O-methoxyethyl RNA analogues, and backbone phosphorothioate, to enhance catalytic efficiency by promoting RNA substrate binding and preventing dimerization of 10–23. These ASO-like DNAzymes cleaved structured RNA targets in long transcripts, showed prolonged intracellular stability, and downregulated mRNA and protein levels of both exogenously transfected eGFP and endogenously elevated oncogenic c-MYC. In colon cancer HCT116 cells, the downregulation of oncogenic c-MYC RNA resulted in cell cycle arrest, reduced proliferation, and increased apoptosis. RACE (rapid amplification of cDNA ends) polymerase chain reaction and Sanger sequencing confirmed precise, site-specific mRNA transcript cleavage with minimal RNase H activation in cells. By merging ASO structural and pharmacokinetic advantages with DNAzyme catalytic versatility, these ASO-like 10–23 variants offer a promising new class of potent gene-silencing agents, representing a significant step toward therapeutic DNAzyme development.

## Graphical Abstract

**Graphical Abstract:**
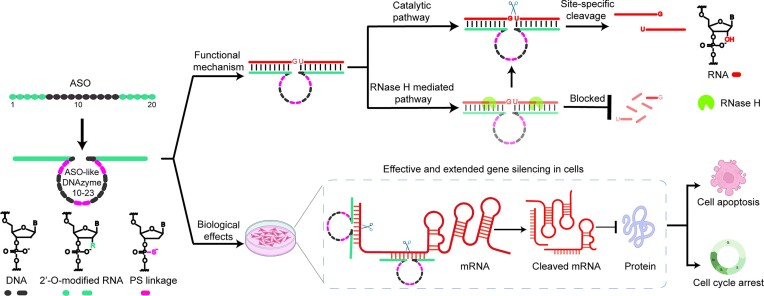

## Introduction

The therapeutic manipulation of RNA to regulate gene expression and interfere with protein synthesis offers a rational avenue for oligonucleotide-based drug discovery. This approach leverages the inherent specificity of Watson–Crick interactions between designed oligonucleotides and target RNA sequences. Particularly noteworthy is the strategy of targeting messenger RNA (mRNA) responsible for disease-causing proteins that were once considered “undruggable”, offering a pragmatic route to therapeutic development [1–3]. Antisense oligonucleotides (ASOs) and small interfering RNA (siRNA) are at the forefront of mRNA-modulating oligonucleotide drugs. ASOs function through steric hindrance, leading to translation arrest, or via RNase H-mediated RNA degradation. On the other hand, siRNA degrades target RNA by engaging the RNA-induced silencing complex [4]. Since the US Food and Drug Administration approved the first ASO drug for silencing human cytomegalovirus (CMV) mRNA in 1998, the advancement of ASO and siRNA therapeutics has been swift. Of the 15 ASO and siRNA drugs granted clinical approval thus far, 5 gained approvals after 2020 [5]. Furthermore, an array of candidates is undergoing clinical trials across different phases [6]. This escalating success underpins the promising potential for developing oligonucleotide-based therapeutics capable of RNA modulation. It also fosters the pursuit of a wider spectrum of RNA-silencing nucleic acid drugs.

In addition to ASOs and siRNAs, RNA-cleaving nucleic acid enzymes represent another class of oligonucleotides with potent RNA-silencing capabilities. These enzymes include ribozymes [7, 8], DNA enzymes (DNAzymes) [9, 10], and the more recently developed xeno nucleic acid enzymes (XNAzymes) [11–15]. Operating upon binding to RNA substrates via Watson–Crick base pairing, RNA-cleaving nucleic acid enzymes exhibit a remarkable degree of sequence specificity in their cleavage activities through their inherent catalytic RNA cleavage activities. They achieve this precision by recognizing nucleotides located at or in proximity to the cleavage site. This stringent nucleotide recognition minimizes off-target effects that often challenge the safety of ASOs and siRNAs. This unique characteristic also empowers these enzymes to distinguish between RNA targets that differ by just one nucleotide. Consequently, they possess the capacity for allele-specific RNA inhibition [14, 16]. Notably, certain XNAzymes have demonstrated the ability to selectively cleave disease-causing mRNA variants bearing single-nucleotide mutations, while leaving the wild-type mRNA unaffected [15]. This underscores a distinct advantage of nucleic acid enzymes over ASOs and siRNAs. Despite these promising attributes, it is noteworthy that no RNA-cleaving enzyme has yet advanced to clinical application. This remains true even for the extensively studied DNAzyme 10–23.

DNAzyme 10–23, often abbreviated as 10–23, is a magnesium (Mg^2+^)-dependent ribonuclease meticulously selected *in vitro* to target a diverse range of therapeutically significant RNAs. Its scope spans *in vitro* contexts, cellular environments, and even phase I/II clinical trials [17–20]. In the presence of 5 mM Mg^2+^, 10–23 achieves a remarkable catalytic efficiency (*k*_cat_/K_M_) ranging between 10^8^ and 10^9^ M^−1^ min^−1^, rivaling its protein counterparts. However, when operating under physiological conditions where the free Mg^2+^ concentration falls below 1 mM, the catalytic efficiency of 10–23 experiences a sharp decline [7, 8]. Although the challenge of Mg^2+^ dependency persists, recent research endeavors to enhance 10–23’s *in vivo* efficacy have primarily concentrated on refining its activity and biostability through chemical modification and evolution. A notable example involves the replacement of substrate binding arms with 2′-deoxy-2′-fluoro arabino nucleic acid (FANA). Additionally, the termini are capped with threose nucleic acid (TNA), capitalizing on FANA’s appropriate binding affinity for RNA and TNA’s nuclease resistance. This strategic configuration, known as X10-23, results in a biologically stable 10–23 variant capable of *in vivo* RNA silencing [16]. In line with this approach, the Chaput laboratory undertook systematic chemical evolution to create another highly modified 10–23 variant, termed Dz 46. By incorporating multiple types of modifications including 2′-OH-modified RNA analogues and phosphorothioate (PS) into both substrate binding arms and catalytic core, Dz 46 exhibited significantly enhanced turnover activity under near-physiological *in vitro* conditions, and displayed the capacity for allele-specific knockdown of the mutant mRNA coding for KRAS G12V oncoprotein within intracellular settings [21]. These examples vividly underscore the potential of comprehensive modification to address the physicochemical requisites essential for 10–23′s therapeutic mRNA-silencing potency *in vivo*.

On the other hand, central to the success of ASO and siRNA therapeutics also lies the strategic incorporation of diverse chemical modifications. These modifications confer distinct physicochemical properties for addressing the comprehensive challenges encountered on the path to effective oligonucleotide-based drugs. These challenges predominantly encompass (i) ensuring chemical stability, (ii) countering nuclease-mediated degradation for improved metabolic stability, (iii) enhancing accessibility to target RNA sites, and (iv) facilitating interactions with proteins and receptors that influence biodistribution, pharmacokinetics (PK), and pharmacodynamics (PD), among others [4, 6, 22–24]. In existing gapmer ASO drugs, RNA-binding-enhancing modifications, typically 2′-O-methyl RNA (OMeRNA), 2′-O-methoxyethyl RNA (MOERNA), or 2′-fluoro RNA (FRNA) in seed regions which localize at the wing regions and are essential for the initial recognition and binding of ASO to the target, alongside PS modifications within the central gap regions or throughout the entire sequence fostering PK and PD requisites, have demonstrated their efficacy in surmounting challenges and propelling oligonucleotides into the realm of therapeutics [5].

Inspired by the molecular blueprint of gapmer ASO drugs, we have engineered ASO-like variants of the DNAzyme 10–23, aiming to replicate the success of ASOs in DNAzymes. This was achieved through targeted catalytic activity-driven introduction of modifications. FRNA, OMeRNA, or MOERNA were incorporated into the substrate binding arms to enhance RNA substrate association. Additionally, multiple PS modifications were introduced across the highly conserved palindromic sequence in the catalytic core to prevent dimerization, which can hinder enzyme activity. The resultant 10–23 variants, designated FRNA-6PS, OMeRNA-6PS, and MOERNA-6PS, exhibited a remarkably improved multiple-turnover rates and augmented RNA substrate accessibility even within highly structured long RNA transcripts under simulated physiological conditions. Moreover, the drug-like attributes instilled by these pivotal modifications, these variants showed prolonged stability and RNA transcript engagement capability in cells. Notably, these attributes were evident in the sustained intracellular mRNA cleavage activity for both exogenously transfected eGFP and endogenously over-expressed c-MYC oncogene with no or minimal activation of RNase H. The potent inhibition of oncogenic c-MYC mRNA by these ASO-like 10–23 variants within colon cancer cell line HCT116, effectively reduced the c-Myc protein expression, which subsequently caused G1 phase cell cycle arrest, decreased proliferation, and increased apoptosis of HCT116 cells. In essence, our work introduces chemically modified 10–23 variants FRNA-6PS, OMeRNA-6PS, and MOERNA-6PS, bearing the core chemistries of ASO drugs, as promising contenders poised to unlock the gene-silencing potential embedded within the RNA-cleaving DNAzyme 10–23.

## Materials and methods

### Materials

All oligonucleotides were ordered from Sangon Biotech (Shanghai), and are listed in Supplementary Tables S1–S3. YM-3 micro centrifugal concentrators were purchased from EMD Millipore. Other molecular biology reagents included PrimeSTAR^®^ Max DNA Polymerase (Takara), FreeZol reagent (Vazyme), HiScript II One Step qRT-PCR SYBR Green Kit (Vazyme), Protease Inhibitor Cocktail (APExBIO, Cat# K1007), Proteinase K Molecular Biology Grade (NEB), T7 RNA Polymerase (NEB), DNase I RNase-free (BBI), RNase Inhibitor (BBI), and *E. coli* RNase H (BBI). Cell biology reagents used in cell culture or manipulation included Dulbecco’s modified Eagle’s medium (DMEM; Gibco, C11995500BT), fetal bovine serum (FBS; HyClone), Hoechst 33258 (BBI), CCK-8 reagent (Vazyme, Cat# A311), Cell Cycle and Apoptosis Analysis Kit (Beyotime, Cat# C1052), and the Annexin V-FITC/PI Apoptosis Detection Kit (Vazyme, Cat# A211). Streptavidin MagPoly Beads were purchased from Smart-Lifesciences (SM017010). Mammalian cell transfection reagent MC3-LNP/SM102-LNP/ALC0315-LNP were purchased from GeneScript (Nanjing).

### Thermal melting measurement by ultraviolet spectroscopy

Samples of 0.3 μM single-stranded 10–23, including wild-type 10–23 (WT), 10–23_4PS, or 10–23_6PS were prepared in 50 mM Tris–HCl buffer (pH 7.5) that contained 200 mM NaCl and 1 mM MgCl_2_. All ultraviolet melting experiments were performed on a SHIMADZU UV-2600i UV-Vis Spectrophotometer with a SHIMADZU S-1700 temperature controller, using a 1-cm-path length quartz cuvette. The samples were scanned from 20°C to 85°C with a temperature increment rate of 1°C/min. The temperature was held at 85°C for 5 min before a reversed scan was performed, from 85°C to 20°C with a rate of 1°C/min. Every scan was monitored at 260 nm. The final melting temperature was obtained by averaging the melting temperatures in the forward and reverse scans.

### *In vitro* kinetic studies

#### Generation of eGFP mRNA transcript for *in vitro* cleavage assay by ASO-like 10–23

Plasmid pscaf-eGFP (+) was subjected to a polymerase chain reaction (PCR) using PrimeSTAR Max DNA Polymerase (Takara, Cat# R045) to introduce a T7 promotor upstream of the coding sequence of eGFP for subsequent *in vitro* transcription. mRNA was then transcribed in a 1× RNAPol reaction buffer, supplemented with 1 mM of each ATP, UTP, GTP, and CTP (BBI, Cat# B500056), 5 mM dithiothreitol (DTT), 2.5 U/ml of pyrophosphatase, inorganic (yeast) (NEB, Cat# M2403S), 1 U/μl of RNase Inhibitor (BBI, Cat# B600478) using 20 ng/μl of the purified DNA amplicon, and 5 U/μl of T7 RNA Polymerase (NEB, Cat# M0251S) at 37°C for 16 h. Transcription was terminated by adding 10 U/ml of RNase-free DNase I (BBI, Cat# B618252) and incubating at 37°C for 15 min. The outputs were purified through phenol–chloroform extraction, ethanol precipitation with LiCl (8 M), further desalting with EMD Millipore YM-3 micro centrifugal device, and quantified for concentration by NanoDrop before *in vitro* kinetic cleavage assays by ASO-like 10–23.

#### Kinetic cleavage reactions for ASO-like 10–23 variants

Single-turnover kinetic cleavage reactions were conducted in 50 mM Tris–HCl buffer (pH 7.5) that contained 200 mM NaCl, 1 mM MgCl_2_, 0.3 μM substrate, and 1.5 μM enzyme at 37°C. Purified enzymes and substrates were annealed in 50 mM Tris–HCl buffer (pH 7.5) by heating for 5 min at 95°C and cooling for 5 min on ice. Reactions were initiated by the addition of NaCl and MgCl_2_ to the reaction. For the determination of the pseudo-first-order rate constant, multiple timepoints were collected by quenching 1 μl of the reaction using 15 μl [15 equiv. (v/v)] of formamide stop buffer [99% deionized formamide, 25 mM ethylenediaminetetraacetic acid (EDTA)] and cooling on ice. Samples were denatured for 10 min at 95°C and analyzed by 15% denaturing polyacrylamide gel electrophoresis (PAGE). Gels were visualized and quantified using an Amersham Typhoon™ laser-scanner platform and quantified using ImageQuant TL v.8.0.2. Values of *k*_obs_ were calculated by fitting the percentage of substrate cleaved and reaction time (min) to the first-order decay equation (1), using Prism 9 (GraphPad):


(1) \begin{eqnarray*}Y = \left( {{{Y}_0} - {{Y}_\infty }} \right) \times \left( {1 - {{e}^{ - {{k}_{{\rm obs}}} \times t}}} \right) + {{Y}_\infty },\end{eqnarray*}

where $Y$ is the percent cleavage at finite time *t*, ${{Y}_0}$ is the percent cleavage at ${{t}_0}$, ${{Y}_\infty }$ is the percent cleavage at an infinite time where the reaction plateaus, and *k*_obs_ is the observed pseudo-first-order rate constant (min^−1^). For kinetic cleavage reactions under stoichiometric and multiple-turnover conditions, substrate concentrations were poised at 2.5 μM, and enzyme concentrations were adjusted to 250 nM.

#### In vitro RNase H activity assays with ASO-like 10–23

All RNase H activity assays were performed in buffer simulating physiological conditions [50 mM Tris–HCl (pH 7.5), 200 mM NaCl] with or without 1 mM MgCl_2_ or 0.1 U/μl of *E. coli* RNase H (BBI, Cat# B110070) at 37°C. A total of 0.3 μM RNA substrate was mixed with 1.5 μM of WT, FRNA-6PS, OMeRNA-6PS, and MOERNA-6PS in a buffer [50 mM Tris–HCl (pH 7.5), 200 mM NaCl] to anneal by heating for 5 min at 95°C followed by cooling for 5 min on ice. Reactions were initiated by adding MgCl_2_ and/or RNase H to the final concentration. Reactions were sampled by quenching 1 μl of the reactions using 15 μl [15 equiv. (v/v)] of formamide containing 25 mM EDTA at 0 and 20 min. Samples were denatured for 10 min at 95°C and analyzed by 15% denaturing PAGE. PAGE gels were visualized using an Amersham Typhoon™ laser-scanner platform and quantified using ImageQuant TL v.8.0.2.

### Intracellular biostability study

#### Generation of HeLa cells lysate for biostability measurement

HeLa cell line was used in this experiment. HeLa cells were cultured in DMEM (Gibco, Cat# C11995500BT) supplemented with 10% FBS, and 1% (1 mg/ml) penicillin and streptomycin and grown at 37°C, 5% CO_2_. At 90% confluence, cells were collected by trypsinization, washed three times with ice-cold phosphate-buffered saline (PBS), lysed with the 10-times volume of the packed cells of HEPES lysis buffer [20 mM HEPES (pH 8.0), 1 mM MgCl_2_, 1% Triton X-100] with Protease Inhibitor Cocktail (APExBIO, Cat# K1007), kept on ice for 30 min with occasional mixing, and then centrifuged at 20 000 × *g* for 20 min at 4°C to clarify the whole cell extract.

#### In vitro incubation with HeLa whole cell lysate

All biostability assays were performed in final volume of 10 μl with about 0.1 × 10^6^ cells’ lysate that contained 10 μM of FRNA-6PS, OMeRNA-6PS, or MOERNA-6PS at 37°C, and then the samples were incubated for 0 and 28 h. Reactions were sampled by quenching 0.5 μl of the reactions using 40 μl [80 equiv. (v/v)] of formamide that contained 25 mM EDTA at timepoints of 0 and 28 h of incubation. Samples were denatured for 15 min at 95°C and analyzed by 18% denaturing PAGE. Gels were visualized using an Amersham Typhoon™ laser-scanner platform and quantified using ImageQuant TL v.8.0.2.

#### Proteinase K digestion

The proteinase K digestion buffer [60 mM Tris, 100 mM EDTA, 400 mM guanidine hydrochloride, 0.1% Triton X-100 (pH 9)] was stored at 4°C. A total of 20 mM DTT was added to aliquots of the buffer immediately before use. Then, 3 μl of digestion buffer and 4.5 μl of proteinase K were added into 9 μl of each sample, and 1.5 μl of water was then added to make the final reaction volume of 18 μl. All samples were mixed well and then incubated at 55°C for 3 h. The reaction was quenched by heating at 95°C for 15 min. A total of 0.9 μl of the heating quenched reactions was sampled into 40 μl of formamide that contained 25 mM EDTA. Samples were then processed using streptavidin magnetic beads. Samples were denatured for 15 min at 95°C and analyzed by 18% denaturing PAGE. Gels were visualized using an Amersham Typhoon™ laser-scanner platform and quantified using ImageQuant TL v.8.0.2.

#### The magnetic bead approach

A proteinase K digestion step was added at the beginning of the extraction protocol. A biotinylated RNA substrate capture strand (18 nt 5′-biotin-rGrArCrGrGrCrArGrCrUrGrCrArGrCrUrCrG-3′) was immobilized on streptavidin magnetic beads, which was used to selectively capture the full length of FRNA-PS, OMeRNA-PS, and MOERNA-PS and their metabolized fragments. Elution buffer containing 10 mM Tris–HCl (pH 7.5) and 1 mM EDTA was prewarmed on a 65°C heat block and a low-salt buffer consisting of 0.15 M NaCl, 20 mM Tris–HCl (pH 7.5), and 1 mM EDTA was chilled on ice. In detail, 100 nmol of biotinylated capture strand was dissolved in 50 μl of wash/binding buffer containing 0.5 M NaCl, 20 mM Tris–HCl (pH 7.5), and 1 mM EDTA. A total of 50 μl of hydrophilic streptavidin-coated magnetic beads was added into a new RNase-free microcentrifuge tube. Then, 50 μl of wash/binding buffer was added to the beads, and they were pipetted to create a suspension. The magnet was applied for ∼30 s, and the supernatant was discarded. Then, 50 μl of biotinylated capture strand was added to the magnetic beads, and the mixture was pipetted. This sample was incubated at room temperature for 10 min with occasional agitation. The magnet was then applied, and the supernatant was discarded. The beads were washed for three times by using 50 μl of wash/binding buffer each time. A total of 18 μl of 2× wash/binding buffer consisting of 1 M NaCl, 40 mM Tris–HCl (pH 7.5), and 2 mM EDTA was added to the samples. They were heated at 95°C for 5 min and then quickly chilled on ice for 3 min for denaturation. The samples were then added into streptavidin-coated magnetic beads immobilized with biotinylated capture strand, pipetted to suspend the particles, and then incubated at room temperature for 20 min with occasional agitation. The magnet was applied, and the supernatant was removed. The beads were then washed with 50 μl of fresh wash/binding buffer for three times, followed by a one-time wash with ice-cold low-salt buffer. A total of 20 μl of prewarmed elution buffer was added followed by pipetting to suspend the beads, and incubated at 65°C for 2 min. The magnet was applied, and the supernatant was transferred to a new tube. Elution was repeated with another 20 μl of fresh elution buffer, and combined with the first elution. A total of 2 μl of elution from each sample was sampled into 40 μl of formamide containing 25 mM EDTA. Samples were denatured for 15 min at 95°C and analyzed by 18% denaturing PAGE. Gels were visualized using an Amersham Typhoon™ laser-scanner platform and quantified using ImageQuant TL v.8.0.2.

### Intracellular eGFP and c-MYC reduction

#### Modification pattern of 10–23 variants used in cellular studies

All the ASO-like 10–23 variants and the WT controls used in cellular studies contained a 3′ inverted dT residue for improved biostability without otherwise specified.

#### Cell lines and mammalian cell culture conditions

HeLa and HCT116 cells were cultured in DMEM supplemented with 10% FBS, 1% (1 mg/ml) penicillin, and 1% (1 mg/ml) streptomycin and grown at 37°C, 5% CO_2_.

#### Transfection of HeLa cells

After 24 h of seeding 0.2 × 10^6^ cells per well, HeLa cells cultured in 12-well plates were transfected with 400 ng of pscaf-eGFP (+) only (negative control) or with 400 ng of pscaf-eGFP (+) and 20, 80, 160, or 320 nM of either version of modified ASO-like 10–23 which targets 69Q-70C or 176V using LNP (lipid nanoparticles) according to the manufacture’s instruction of SM102 LNP. For negative controls, the volume of LNP reagent used for each well was the same as those with DNAzymes to ensure the same transfection conditions in the control and experimental samples. At 24, 48, and 72 h post-transfection, cells were harvested and subjected to total RNA extraction.

#### Transfection of HCT116 cells

For cleavage site screening assays, 24 h after seeding of 2.4 × 10^6^ cells per well, HCT116 cells cultured in six-well plates were transfected with LNP only as a negative control or with 320 nM of FRNA-6PS targeting eight different cutting sites, denoted as Myc-1–Myc-8, using LNP. At 24 h post-transfection, cells were harvested and subjected to total RNA and protein extraction. For the activity of all three ASO-like 10–23 targeting Myc-1, after 24 h of seeding of 2.4 × 10^6^ cells per well, HCT116 cells cultured in six-well plates were transfected with 320 nM of each ASO-like10–23 variant, with LNP only as negative control. At 18 or 24 h post-transfection, cells were harvested and subjected to total RNA and protein extraction.

#### Cell imaging

At 24, 48, or 72 h post-transfection, co-transfected HeLa cells were subjected to live imaging using an Olympus CKX53 invert microscope with a 10× objective and pE-300 Lite white light LED illumination system with a 488-nm filter. After imaging, the cells were subjected to RNA extraction.

#### Confocal imaging of Cy5-labeled ASO-like 10–23 for biostability assay in HeLa cells

Approximately 25 000 HeLa cells were seeded in four-well 35-mm glass-bottomed culture dishes and incubated overnight. Then, 160 nM of 5′ Cy5-labeled ASO-like 10–23 variants (FRNA-6PS, OMeRNA-6PS, and MOERNA-6PS) were transfected to HeLa cells using LNP following the SM102 LNP transfection protocol. After incubation for 28, 56, and 74 h, the cell nuclei were stained with 2 μg/ml Hoechst 33342 (BBI, Cat# 607302) at 37°C for 15 min, then washed two times with 1× Dulbecco’s Phosphate-Buffered Saline (DPBS). The cells were fixed with 4% paraformaldehyde in the dark at room temperature for 10 min. The cell images were acquired with a Nikon Laser Scanning Confocal Microscope.

#### RNA isolation and RT-qPCR

For all samples, RNA was extracted using the FreeZol reagent (Vazyme, Cat# R711) according to the manufacturer’s instructions. To quantify the copy number of the eGFP or c-MYC transcript in the presence or absence of ASO-like 10–23, RNA was subjected to reverse transcription and quantitative PCR (RT-qPCR) analysis using HiScript II One Step RT-qPCR SYBR Green Kit (Vazyme, Cat# Q221) on a Roche LightCycler 96 system. Specific primers to interrogate eGFP, c-MYC, and glyceraldehyde 3-phosphate dehydrogenase (GAPDH; loading control) transcripts as well as for the qPCR standards are listed in Supplementary Table S2. For each experimental sample, three technical replicates and two biological replicates were performed. The relative mRNA copy number of the target transcripts was calculated by comparing the levels of target gene (eGFP and c-MYC) sequences with a corresponding scaling factor derived from the loading control GAPDH in the sample, and the final result was shown as a ratio of these genes.

#### RACE PCR

5′ RACE (rapid amplification of cDNA ends) PCR was performed using a Template Switching RT Enzyme Mix (NEB, Cat# M0466) according to the manufacturer’s instructions. Specific template-switching oligo (TSO) and primers are listed in Supplementary Table S3. Complementary DNA (cDNA) was synthesized from 400 ng of total RNA, purified from HeLa cells co-transfected with 400 ng CMV-eGFP plasmid and 320 nM of each ASO-like 10–23 variant, in a 10-μl reverse transcription-template switching (RT-TS) reaction using a random primer mix (NEB, Cat# S1330S) and 0.35 μM of TSO. The reaction was incubated at 42°C for 90 min. The RT-TS reaction was diluted two-fold with diethylpyrocarbonat (DEPC)-treated water, and 4 μl of the diluted cDNA was used in a subsequent 100-μl PCR reaction.

cDNA was amplified using a two-step nested PCR strategy with PrimeSTAR Max DNA Polymerase (Takara, Cat# R045) for RACE1 PCR and Taq DNA Polymerase (NEB, Cat# M0267S) for RACE2 PCR. The first PCR used 0.5 μM forward primer TS1 and 0.5 μM reverse primer eGFP-R-RACE-1 with touch-down PCR. The cycling conditions were as follows: 98°C for 30 s, five cycles of (98°C for 10 s and 72°C for 30 s), five cycles of (98°C for 10 s and 70°C for 30 s), 35 cycles of (98°C for 10 s, 68°C for 15 s, and 72°C for 30 s), and a final extension at 72°C for 5 min. Products were analyzed on 2% agarose gels stained with EB (MACKLIN, Cat# E854573) and purified using a gel extraction kit (AidLab, Cat# DR0101).

The RACE2 PCR used 1 μl of purified product as a template in a 50-μl reaction with 1 μM forward primer TS2 and 1 μM reverse primer 176V Q-rev. Cycling conditions were as follows: 95°C for 3 min, 25 cycles of (95°C for 15 s, 55°C for 15 s, and 72°C for 30 s), and a final extension at 72°C for 5 min. After 2% agarose gel purification, products were cloned using a TOPO TA cloning kit (Sangon, Cat# B522215) according to the manufacturer’s instructions. Selected colonies were cultured overnight, and bacterial cultures were used for Sanger sequencing (Genewiz).

#### Protein isolation and western blot analysis

At 18 or 24 h post-transfection, HCT116 cells were collected via trypsin digestion, divided in a 2:3 ratio (2/5 for RNA isolation and the remainder for protein extraction), and centrifuged at 2500 × *g* for 5 min at 4°C. Cell pellets for protein isolation were resuspended in 50–100 μl (10 × pellet volume) of Tris lysis buffer [20 mM Tris–HCl (pH 8.5), 150 mM NaCl, 10 mM MgCl_2_, 0.5% Triton X-100, 1 mM EDTA] supplemented with 10 μl of Protease Inhibitor Cocktail (APExBIO, Cat# K1007) and 10 μl of Phosphorylase Inhibitor Complex I (Sangon, Cat# C500017) per milliliter of buffer. Cells were shaken at 4°C for 30 min. Total protein lysate was cleared by centrifugation at 14 800 rpm for 30 min at 4°C. Protein concentration was determined using a BCA assay kit (Sangon, Cat# C503021) according to the manufacturer’s instructions at 37°C for 30 min. Absorbance at 562 nm (A562) was measured using a Tecan Spark microplate reader. A standard curve was generated in Microsoft Excel, plotting average A562 values of standards against protein concentrations. Sample protein concentrations were calculated from the standard curve and adjusted for dilution.

Sodium dodecyl sulfate–polyacrylamide gel electrophoresis (SDS–PAGE) analysis was performed with 10 μg of total protein per well, resolved in a 10-well 5% stacking and 10% separating SDS–PAGE gel. Proteins were transferred to a 0.45-μm nitrocellulose membrane using a wet transfer method with transfer buffer [25 mM Tris, 190 mM Glycine (pH 8.2–8.4), 20% methanol]. Membranes were blocked with 5% nonfat milk in 1× tris buffered saline with tween 20 (TBST) [150 mM NaCl, 50 mM Tris–HCl (pH 7.6), 0.1% Tween-20] for 1 h at room temperature, then probed overnight at 4°C with c-Myc Rabbit mAb (ABclonal, Cat# A19032) at 1:1000 dilution or GAPDH rabbit pAb (Sangon, BBI, Cat# D110016) at 1:5000 dilution in 1× TBST. Blots were washed three times with 1× TBST at room temperature (10 min for each wash), then incubated with HRP-conjugated goat-anti-rabbit IgG (Sangon, Cat# D110058) in fresh 5% nonfat milk blocking buffer (1:5000 dilution) for 1 h at room temperature. Finally, blots were washed three times with 1× TBST at room temperature again (10 min each wash) and imaged using ECL Chemiluminescent Substrate (Sangon, Cat# C500044) on an Amersham ImageQuant 800 platform (Cytiva), and bands quantified using ImageQuant TL v.8.0.2.

#### CCK-8 cell viability analysis

After 24 h of seeding 5 × 10^4^ cells per well, HCT116 cells cultured in 96-well plates were transfected with LNP only as negative control, or 320 nM of every version of ASO-like 10–23 targeting the Myc-1 cutting site using LNP. At 24 h post-transfection, 10 μl of CCK-8 reagent (Vazyme, Cat# A311) was added to each well and incubated at 37°C for 45 min. The absorbance at 450 nm (A450) was measured using a Tecan Spark microplate reader. Normalized viability was calculated as follows: viability = [Abs (sample) – Abs (background)]/[Abs (max) – Abs (background)], where Abs (max) was the LNP-only control and Abs (background) was the medium-only control. Each experiment included three biological replicates and five technical replicates.

#### Cell cycle analysis

After 24 h of seeding 2.4 × 10^6^ cells per well, HCT116 cells cultured in six-well plates were transfected with LNP only as negative control, 320 nM of each version of ASO-like10–23 targeting the Myc-1 cutting site using LNP. At 18 or 24 h post-transfection, HCT116 cells were collected via trypsin digestion and centrifuged at 2500 × *g* for 5 min at 4°C. The cell pellet was resuspended in 1 ml of cold 70% ethanol and fixed overnight at 4°C. Fixed cells were washed twice with 1× PBS and treated with the Cell Cycle and Apoptosis Analysis Kit (Beyotime, Cat# C1052) according to the manufacturer’s instructions. Propidium iodide (PI)-stained cells were analyzed for cell cycle distribution (G1, S, and G2 phases) using an Attune NxT flow cytometer (Thermo Fisher). Data were analyzed with FlowJo_v10.10.0 software with cell doublets excluded. Each experiment included three biological replicates and three technical replicates.

#### Apoptosis analysis

Cell apoptosis was assessed using the Annexin V-FITC/PI Apoptosis Detection Kit (Vazyme, Cat# A211). HCT116 cells were seeded in 96-well plates at a density of 5 × 10^4^ cells per well for FRNA-6PS and OMeRNA-6PS, and 7.5 × 10^4^ cells per well for MOERNA-6PS. After 24 h, cells were transfected with LNP only (negative control) or 320 nM of each ASO-like 10–23 targeting Myc-1. The LNP–oligo mixture was diluted with 100 μl of medium supplemented with either 1 μl of Annexin V-FITC or 3 μl of PI staining solution per 600 μl of medium per well. The plates were incubated in the Incucyte S3 Live-Cell Analysis System (Sartorius) for 48 h, with bright-field and green or red fluorescence images captured every 6 h.

## Results

### Catalytic activity-directed modification of the catalytic core by introducing PS linkages

**Figure 1.**
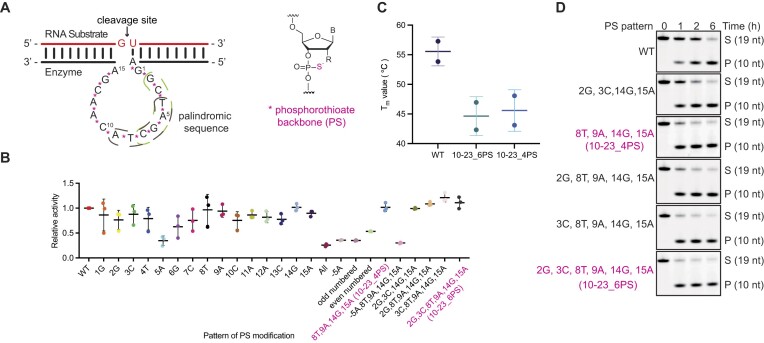
Catalytic activity-directed modification of the catalytic core of 10–23 by introducing internucleoside PS linkages. (**A**) Left panel: The complex formed between 10–23 and an RNA substrate through Watson–Crick base pairing. The GU dinucleotide cleavage junction within the RNA substrate is indicated by an arrow. The two six-letter palindromic sequences are highlighted. Right panel: The chemical structure of the PS linkage within the backbone structure. (**B**) Catalytic activity-directed mapping of the catalytic core of 10–23 through PS substitutions. The graph depicts the relative substrate cleavage normalized to WT. Error bars represent ±standard deviation (s.d.) of the mean for three independent replicates. (**C**) Thermal melting study of homoduplexes formed by 10–23. Thermal melting experiments were conducted using homoduplexes formed by 10–23 containing multiple PS linkages within the catalytic core. Measurements were performed in a buffer containing 50 mM Tris–HCl (pH 7.5), 2.5 mM MgCl_2_, and 200 mM NaCl. Each variant of 10–23 was maintained at 3 μM, and absorbance was recorded at 260 nm. The reported *T*_m_ values are the averages of three separate forward and reverse measurements. Error bars indicate ±s.d. (**D**) Representative PAGE gels illustrating RNA cleavage activity of PS-modified 10–23 under multiple-turnover conditions with 2.5 μM substrate and 250 nM enzyme. All cleavage reactions were performed in 50 mM Tris–HCl (pH 7.5) containing 1 mM MgCl_2_ and 200 mM NaCl at 37°C. S, substrate; P, cleavage product.

Previous X-ray crystallography [25] and the latest nuclear magnetic resonance (NMR) study [26] of the structure and catalytic mechanism of the 10–23 enzyme revealed that a significant portion of the 10–23 sequence tends to form homoduplexes. This is attributed to the presence of two six-letter palindromic sequences, namely 5′-GCTAGC-3′ and 5′-TAGCTA-3′ (Fig. 1A). The self-dimerization of these sequences renders the enzyme catalytically inept. While a single nucleotide mutation disrupting the palindromic sequences could prevent homodimer formation, such mutations might result in a substantial reduction of activity, given the high conservation of the catalytic core of 10–23 [27] . To address this challenge, we conceived the idea of introducing PS modifications into the catalytic core. This concept builds upon the well-established fact that the incorporation of PS modifications with mixed stereochemistry into DNA leads to a decrease in the melting temperature (*T*_m_) of duplexes formed with their reverse complementary strands [28]. Additionally, PS modifications have been known to confer various biological effects, making them one of the key modifications for ASO drugs [29, 30]. We initiated our study by systematically replacing each 5′ PO linkage with a PS linkage throughout the catalytic core (1G to 15A), generating a series of fifteen 10–23 variants. Upon subjecting these variants to short RNA substrate cleavage assays, most of them exhibited activity levels similar to or slightly compromised compared with the WT. Notably, the 5-position adenine (5A) PS substitution was an exception, showing only ∼30% of the activity relative to WT (Fig. 1B). Although definitive structural data to explain this phenomenon are lacking, it is plausible that the specific phosphate group connecting 4T and 5A located within the metal-ion-binding site of the catalytic core, as suggested by a time-resolved NMR structural study, closely involved in metal ion binding. Substituting PS for PO resulted in decreased Mg^2+^-binding affinity, leading to a subsequent drop in catalytic activity. Subsequently, we aimed to identify a catalytic core modification pattern with the maximum extent of PS substitutions spanning the palindromic sequences, while still maintaining the catalytic activity of 10–23. From a range of evaluated combination patterns, we identified five variant catalytic cores carrying four–six PS substitutions. Importantly, these variants exhibited improved catalytic activity compared with the WT (Fig. 1B and D). Consistent with our expectations, the *T*_m_ values of the homoduplexes exhibited a PS linkage number-dependent decrease of ∼10°C when four or six PS substitutions (denoted as 10–23_4PS and 10–23_6PS, respectively) were present within the core (Fig. 1C and Supplementary Fig. S1). Subsequent chemical modifications and studies were conducted based on 10–23_6PS as it demonstrated both desirable catalytic activity and a reduced likelihood of dimerization, unless otherwise stated.

### Enhancing the catalytic activity of 10–23_6PS through RNA analogue substitutions in substrate-binding arms

**Figure 2.**
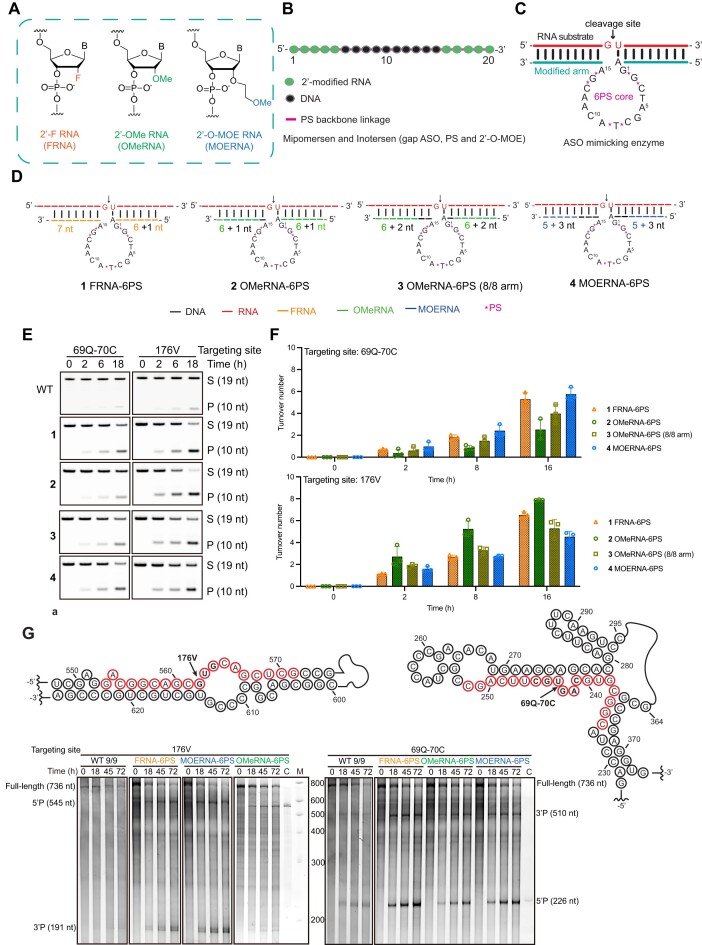
*In vitro* catalytic activity of ASO-like 10–23 molecules. (**A**) Chemical structures of 2′-modified RNA used in gapmer ASO drugs. Abbreviations: FRNA (2′-fluoro RNA/2′-F RNA), OMeRNA (2′-O-methyl RNA/2′-OMe RNA), and MOERNA (2′-O-methoxyethyl RNA/2′-O-MOE RNA). (**B**) Modification pattern of gapmer ASO. (**C**) Modification pattern of ASO-like 10–23 (bottom strand), combining 6PS linkages in the catalytic core and RNA-binding-enhancing chemical modifications employed by ASO drugs in the substrate binding arms. (**D**) Optimization of the modification pattern of substrate-binding arms of 10–23 by FRNA (1), OMeRNA (2 and 3), and MOERNA (4). (**E**) Representative PAGE gels showing the activity of each modification pattern of ASO-like 10–23 depicted in panel (B) for targeting short RNA substrates derived from two different sites of the mRNA of eGFP. All cleavage assays were performed under multiple-turnover conditions with 2.5 μM substrate and 250 nM enzyme in 50 mM Tris–HCl (pH 7.5) containing 1 mM MgCl_2_ and 200 mM NaCl at 37°C. S, substrate; P, cleavage product. (**F**) Quantitative analysis of the turnover activity shown in panel (E). Top graph: Cleavage activity of construct 1–4 on RNA substrate denoted as 69Q-70C. Bottom graph: Cleavage activity of construct 1–4 on RNA substrate denoted as 176V. Error bars denote ±s.d. of the mean for *n* = 3 independent replicates. (**G**) *In vitro* activity of FRNA-6PS, OMeRNA-6PS, and MOERNA-6PS cleaving the full-length structured mRNA coding eGFP under simulated physiological conditions. Top panel: The sequences and secondary structures of targeting sites denoted as 176V (left) and 69Q-70C (right). The RNA sequences involved in the recognition and cleavage by variant 10–23s are colored, with the arrows designating the exact cleavage sites within the codons (highlighted in bold) of 176V and 69Q-70C. Bottom panel: Representative PAGE gels showing the multiple-turnover cleavage activity of FRNA-6PS, OMeRNA-6PS, and MOERNA-6PS in comparison to WT containing substrate binding arms of 9 nt (WT 9/9). All the assays were performed under multiple-turnover conditions with 2.5 μM of substrate and 250 nM of enzyme in 50 mM Tris–HCl (pH 7.5) containing 1 mM MgCl_2_ and 200 mM NaCl at 37°C.

In the realm of approved ASO and siRNA drugs, the strategic integration of 2′-O-modified RNA analogues (depicted in Fig. 2A) within the seed region has proven pivotal for achieving high-affinity RNA binding. This, in turn, optimizes target RNA accessibility, particularly in the context of complex, structured long RNA transcripts, effectively mitigating off-target effects (as illustrated in Fig. 2B). Building on this successful design, we transposed its principles onto the 10–23 system. Our approach involved replacing nucleotides within the substrate-binding arms flanking the catalytic core that already bore six PS substitutions (10–23_6PS) with three distinct RNA analogues, 2′-F, 2′-OMe, and 2′-OMOE RNAs (Fig. 2C). These modifications were chosen due to their established utility in ASO drugs approved and in clinical trials, along with their well-characterized chemophysical and biological properties, and the feasibility of scalable synthesis [31].

**Figure 3.**
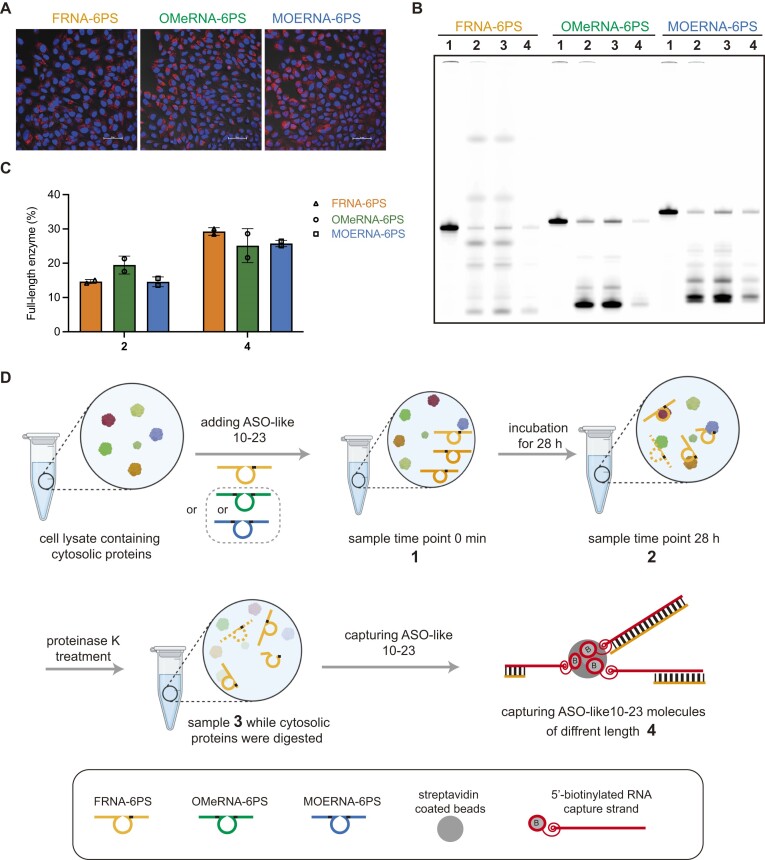
The intracellular stability and availability of FRNA-4PS, OMeRNA-6PS, and MOERNA-6PS. (**A**) Confocal imaging of intracellular oligomers. Confocal images were taken 28 h after transfecting HeLa cells with 160 nM of 5′-Cy5 dye-labeled enzyme variants. All three types of molecules exhibited significant signals within the cells. Nuclei were labeled using Hoechst 33258. Scale bar, 100 μm. (**B**) Representative PAGE analysis of samples 1–4 obtained from treatment of FRNA-6PS, OMeRNA-6PS, and MOERNA-6PS, respectively. (**C**) The quantitatively assessed percentage of free, full-length FRNA-6PS, OMeRNA-6PS, or MOERNA-6PS not bound to any protein (2) or recovered through the reverse complementary capture RNA strand (4) post proteinase K treatment. (**D**) Schematic representation illustrating the assessment of the biostability of FRNA-6PS, OMeRNA-6PS, and MOERNA-6PS under simulated intracellular conditions. 100 nM of FRNA-4PS, OMeRNA-6PS, or MOERNA-6PS labeled with 5′-Cy5 were incubated in a buffer [20 mM HEPES (pH 8.0), 1 mM MgCl_2_, 1% Triton X-100] enriched with cell lysate containing cytosolic proteins. Samples were collected at timepoint 0 h (1) and 28 h after incubation, before (2), and after (3) proteinase K treatment. FRNA-4PS, OMeRNA-6PS, or MOERNA-6PS molecules, whether full-length or partially digested by cytosolic proteins, were captured using a 5′-biotinylated reverse complementary RNA strand. Duplexes formed were subsequently isolated using streptavidin-coated magnetic beads (4).

**Figure 4.**
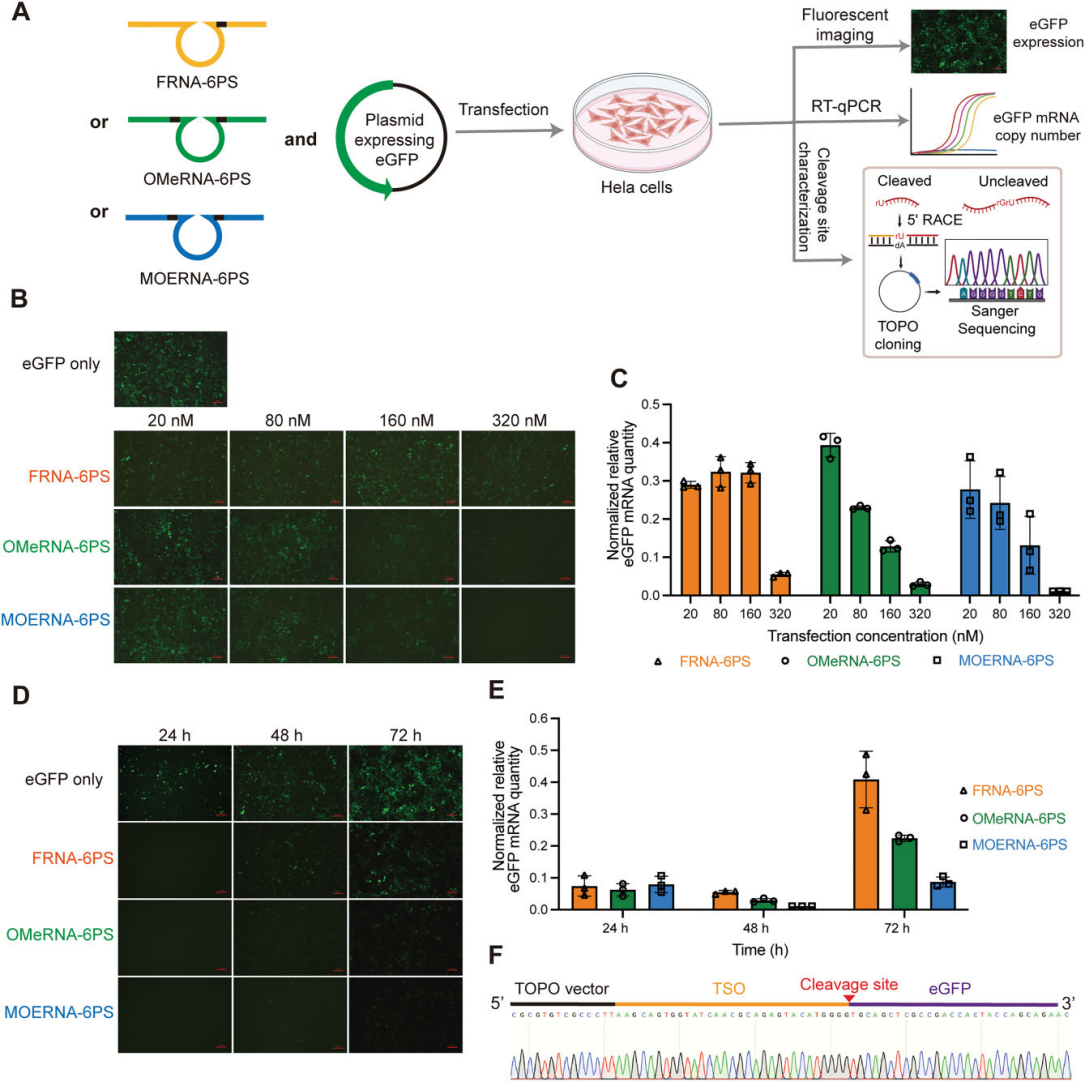
eGFP inhibition activity of FRNA-6PS, OMeRNA-6PS, or MOERNA-6PS in HeLa cells. (**A**) Schematic representation of using FRNA-6PS, OMeRNA-6PS, and MOERNA-6PS molecules for the intracellular inhibition of exogenously transfected eGFP. Fluorescent imaging of cells was performed to qualitatively assess the inhibitive effect to the level of eGFP expression. RT-qPCR was performed to quantitatively assess the catalytic reduction of eGFP mRNA copy number. 5′ RACE PCR reactions templated by cDNA derived from HeLa cells 24 h after transfections with 320 nM of each variant were subjected to Sanger sequencing to characterize the cleavage site on the mRNA of eGFP. (B, C) Dose-dependent eGFP inhibition. (**B**) Fluorescent cell images collected prior to harvesting the cells at 48 h post-transfection. Scale bar, 100 μm. (**C**) RT-qPCR quantification of DNA-free total RNA isolated 48 h post-transfection using eGFP-specific and GAPDH loading control primers. (D, E) Time-dependent eGFP inhibition when cells were transfected with 320 nM of each version of enzyme. (**D**) Fluorescent cell images collected prior to harvesting the cells at 24, 48, and 72 h post-transfection. Scale bar, 100 μm. (**E**) RT-qPCR analysis of DNA-free total RNA isolated at different times post-transfection using eGFP-specific and GAPDH loading control primers. Two biological replicates and three technical replicates were collected for each condition, with one representative biological replicate shown in panels (C) and (E). The mRNA copy number of eGFP relative to that of GAPDH for cells co-transfected by eGFP plasmid and FRNA-6PS, OMeRNA-6PS, or MOERNA-6PS was further normalized to that for cells transfected with only eGFP plasmid. Error bars are ±s.d. of the mean for *n* = 3 technical replicates. (**F**) 5′ RACE PCR and Sanger sequencing revealed the sequence derived from the 3′ cleavage product of ASO-like 10–23 variants mediated cleavage (cleavage site, indicated by the arrow) of eGFP mRNA (eGFP), appending to a RACE TSO.

The introduction of RNA analogues into the substrate-binding arms triggers considerable alterations within the duplex formed between 10–23 and the RNA substrate. Given this, careful consideration was given to preserve the conformation proximal to the cleavage site, to achieve which, we performed comprehensive nucleotide substitution-and-activity studies to obtain the optimal modification patterns of the substrate-binding arms. For incorporating FRNA bearing the least bulky 2′-F substitution, we spared only the internal dA residue within the substrate-binding arm that interacts with the rU in the r(GU) dinucleotide cleavage site (Fig. 2D-1). In parallel, for OMeRNA characterized by a moderately bulky 2′-O-methyl group, we retained one or two internal DNA residues in either substrate-binding arm (Fig. 2D-2 and D-3). Lastly, for MOERNA bearing the most substantial 2′-O-methoxyethyl group, we left three internal DNA residues unaltered (Fig. 2D-4). Meantime, these substitutions yielded distinctive substrate RNA affinities, with FRNA exhibiting the highest (Δ*T*_m_ of ∼2.5°C per nucleotide) [32, 33], followed by MOERNA and OMeRNA (Δ*T*_m_ of 0.9°C–1.7°C per nucleotide) [34]. To achieve an optimal kinetic effect with a balance between accelerating enzyme–substrate association pre-catalysis and preventing drastic inhibition to cleavage product release post-catalysis which can be entailed by careful control over the number of RNA analogue substitutions, we conducted systematic evaluations employing two 19-nt short RNA substrates derived from the eGFP mRNA transcript. Designated as 69Q-70C and 176V based on the amino acids encoded by the mRNA sequences spanning the cleavage site (Supplementary Fig. S2), these substrates facilitated the assessment of catalytic activities of modified 10–23 variants. These analyses encompassed substrate-binding arms ranging 7–9 nt on each side, under both pseudo-first-order (single-turnover; Supplementary Fig. S3) and multiple-turnover conditions simulating physiological pH (50 mM Tris–HCl, pH 7.5) and Mg^2+^ concentration (1 mM). In comparison to the original WT with substrate-binding arms ranging 7–9 nt, the FRNA-6PS variant with 7-nt binding arms (Fig. 2D-1), OMeRNA-6PS with 7-nt and 8-nt binding arms [referred to as OMeRNA-6PS and OMeRNA-6PS (8/8 arm); Fig. 2D-2 and D-3], and MOERNA-6PS with 8-nt binding arms (Fig. 2D-4) displayed substantially heightened multiple-turnover catalytic activities. Intriguingly, this catalytic performance was correlated with the target RNA sequence, as all three variants exhibited slightly faster turnover on substrate 176V compared with 69Q-70C (Fig. 2E and F).

Notably, these enhanced catalytic activities were recapitulated when FRNA-6PS, OMeRNA-6PS, and MOERNA-6PS were applied to cleave the full-length 736-nt eGFP mRNA transcript. Operating under simulated physiological conditions [50 mM Tris–HCl (pH 7.5), 1 mM Mg^2+^, and 37°C], these modified enzymes poised at 250 nM, executed precise cleavage at the sites corresponding to both 176V and 69Q-70C, the deeply entrenched cleavage sites within intricate secondary structures of the eGFP transcripts that were in 10-fold excess (2.5 μM), and generated products of the correct length. In stark contrast, the original WT with 9-nt substrate-binding arms exhibited drastically weakened activity at cleavage site 69Q-70C and barely discernible activity at cleavage site 176V. Surprisingly, while generally stronger cleavage was observed at site 176V when dealing with 19-nt short substrates, FRNA-6PS, OMeRNA-6PS, and MOERNA-6PS consistently exhibited superior performance at cleavage site 69Q-70C when tasked with cleaving the 736-nt eGFP transcript (Fig. 2G). This phenomenon underscores the pivotal role of target site selection for optimal gene-silencing efficacy using such nucleic acid agents. The substantial enhancement in catalytic activity for cleaving structured, lengthy RNA transcripts under simulated physiological conditions *in vitro* positions FRNA-6PS, OMeRNA-6PS, and MOERNA-6PS as promising candidates for *in vivo* RNA-silencing applications.

### FRNA-6PS, OMeRNA-6PS, and MOERNA-6PS functioned via intrinsic catalytic activity with minimal activation of RNase H

Questions have arisen regarding the mechanisms underlying the intracellular and *in vivo* target RNA silencing achieved by 10–23 and its variants. Specifically, there is a debate about the RNA-silencing activity should be attribute to intrinsic catalytic activity or RNase H-mediated antisense activity [35]. Considering that cellular RNase H recognizes DNA-based substrate-binding arms as ASOs, we hypothesized that configuring substrate-binding arms primarily with RNA analogues would suppress RNase H-mediated RNA degradation, which lacks sequence specificity, while maintaining intrinsic catalytic cleavage at the r(GU) dinucleotide. To investigate this hypothesis, we assessed the cleavage activity of FRNA-6PS, OMeRNA-6PS, and MOERNA-6PS on a 19-nt RNA substrate in the presence or absence of RNase H activity, controlled by the presence or absence of 1 mM Mg^2+^, the required cofactor by RNase H. Denaturing analytical PAGE gels revealed that FRNA-6PS autonomously cleaved the RNA substrate site specifically via intrinsic catalytic activity (asterisks in Supplementary Fig. S4B), with no detectable RNase H-mediated RNA degradation. While OMeRNA-6PS primarily operated through intrinsic catalytic activity, minor RNase H-mediated cleavage products were observed. In contrast, MOERNA-6PS triggered more significant antisense mechanism-based RNA degradation, yet intrinsic catalytic cleavage remained the main mechanism (Supplementary Fig. S4B). The extent of RNase H activity suppression appeared related to the portion of RNA residues that was in hybridization with the RNA analogues introduced into the substrate-binding arms, aligning with our rationale for using RNA analogue substitutions to mitigate antisense mechanism-based activity of 10–23. In contrast, WT and an inactive version with a catalytic core mutation (5A-to-G) both prompted substantial RNase H-mediated RNA degradation. This observation aligns with reports of RNase H-mediated RNA degradation in cells transfected with WT or inactive 10–23 versions [35]. The near elimination of RNase H-mediated substrate cleavage (as seen with FRNA-6PS) or its significant suppression (as with OMeRNA-6PS and MOERNA-6PS), coupled with the dramatic enhancements in turnover rates and accessibility to target sequences concealed within complex RNA structures underscores their distinct capability to accurately discern RNA substrates that differ by a mere single nucleotide with significantly attenuated risk of “off-target” effects arising from interference by the antisense mechanism.

### Intracellular stability and availability of FRNA-6PS, OMeRNA-6PS, and MOERNA-6PS

Enhancing resistance to nucleases and metabolic instability is a paramount concern for oligonucleotide therapeutics. While significant progress has been made in validating the enhanced biostability of oligonucleotides modified with diverse nucleotide analogues *in vitro* using biologically relevant agents such as nucleases, human serum, and liver microsomes, our understanding of their behavior intracellularly remains limited [36, 37]. The intracellular residence time of oligonucleotides surviving nuclease degradation and their accessibility for catalytic activity within the complex milieu of intracellular proteins have yet to be thoroughly investigated. Addressing these critical aspects is essential for guiding the intracellular application of FRNA-6PS, OMeRNA-6PS, and MOERNA-6PS. To address these aspects comprehensively, we employed both qualitative and quantitative approaches.

Initially, we qualitatively assessed the intracellular fate of FRNA-6PS, OMeRNA-6PS, and MOERNA-6PS by tracking their intracellular fluorescent signals using confocal imaging. Cy5-dye-labeled oligonucleotides were transfected into cells, resulting in intense cytoplasmic signals. These signals were mainly localized in the cytoplasm, indicative of their escape from endosomes, a process thought to occur within 2–6 h post-transfection (Fig. 3A). Over time, the intracellular intensity of Cy5 signals diminished as compared with the signal of Hoechst 33258 for nuclei staining (shown in blue), which was revealed by cell images captured at 52 and 74 h post-transfection (Supplementary Fig. S5). Nonetheless, PAGE gel analysis of FRNA-6PS, OMeRNA-6PS, and MOERNA-6PS incubated in cell lysates simulating intracellular conditions up to 74 h showed more than one-third of full-length populations observed at 28 h post-transfection were still remained at timepoint 72 h (Supplementary Fig. S6), which corroborated the observed decrease of intracellular oligomer signal over time, which was presumably attributed to both cytosolic protein-caused degradation and cell division-caused dilution.

We next conducted a quantitative analysis to investigate the potential intracellular substrate engagement capabilities of FRNA-6PS, OMeRNA-6PS, and MOERNA-6PS molecules. These molecules were subjected to incubation under simulated intracellular conditions. Specifically, FRNA-6PS, OMeRNA-6PS, and MOERNA-6PS were incubated for 28 h in a cell lysate containing all cytosolic proteins, which was prepared from HeLa cells. We obtained characteristic samples at various timepoints: 0 min (1), 28 h post-incubation before proteinase K treatment (2), and 28 h post-incubation after proteinase K treatment (3). Following the proteinase K, full-length and fragmented forms of FRNA-6PS, OMeRNA-6PS, and MOERNA-6PS that were presented in free form and released by digested proteins were captured using a reverse complementary RNA strand that was modified with a 5′-biotin group. This modification enabled solid-phase extraction using streptavidin-coated magnetic beads (4). The overall workflow is depicted in Fig. 3D. The samples were resolved using denaturing analytical PAGE gel (Fig. 3B), revealing that a population of the full-length oligonucleotides survived enzymatic degradation and cellular protein adsorption accounting for 15%–20% of the total oligonucleotide species in sample 2 (Fig. 3C). This observation indicated a considerable presence of free-form FRNA-6PS, MOERNA-6PS, and OMeRNA-6PS capable of effectively engaging in intracellular RNA cleavage reactions. In sample 4, where proteinase K treatment released the oligonucleotides bound to cellular proteins, the relative amount of full-length oligonucleotides increased by ∼15%, ∼10%, and ∼5% for FRNA-6PS, MOERNA-6PS, and OMeRNA-6PS, respectively. The varied extents of interference arising from intracellular proteins (with the order FRNA-6PS > MOERNA-6PS > OMeRNA-6PS) suggested that the intracellular functional efficacy of these molecules might differ from their catalytic performance observed *in vitro*. This insight provides a basis for designing dosage and interpreting activities when subjecting these molecules to intracellular or *in vivo* mRNA inhibition activities. Sample 1 solely served as an electrophoretic size marker for locating the free full-length population in each oligonucleotide species.

### Intracellular inhibition of constitutively expressed exogenous eGFP

We further explored the efficacy of FRNA-6PS, OMeRNA-6PS, and MOERNA-6PS within cultured HeLa cells for silencing the expression of exogenously transfected eGFP, which was constitutively active. To achieve this, HeLa cells were co-transfected with a plasmid expressing eGFP under the control of a human CMV promoter and 10–23 variants of FRNA-6PS, OMeRNA-6PS, or MOERNA-6PS that targeted the cleavage site of 176V. Fluorescent cell images were captured before harvesting the cells for quantifying eGFP mRNA copy numbers using quantitative reverse-transcription PCR (qRT-PCR) (Fig. 4A). In comparison to cells transfected solely with the eGFP plasmid, the co-transfection of the eGFP plasmid with FRNA-6PS, OMeRNA-6PS, or MOERNA-6PS, at concentrations ranging from 20 to 320 nM, resulted in varying degrees of reduction in eGFP signals after 48 h (Fig. 4B and Supplementary Fig. S7A). qRT-PCR analysis confirmed that the decrease in eGFP signal was attributed to a reduction in the mRNA template copy number for eGFP (Fig. 4C). Notably, at a concentration of 320 nM, all three constructs resulted in a remarkable reduction of over 90% in eGFP mRNA copy numbers compared with cells transfected solely with the eGFP plasmid. While each of the three constructs still exhibited mRNA reduction of >40% at lower concentrations, it is important to note that the dose-dependent effect on eGFP inhibition was either not observed (for RNA-6PS below 160 nM) or not as significant as OMeRNA-6PS (for MOERNA-6PS below 80 nM). This observation led us to postulate that at lower concentrations, the interactions between the more protein-affinitive FRNA-6PS and MOERNA-6PS with cellular proteins might have exerted relatively large effects on their effective concentrations that can engage in mRNA cleavage. Despite of the potential protein-binding interference, owing to the intrinsically higher catalytic activity of MOERNA-6PS on cleaving the site of 176V of the eGFP whole transcript (Fig. 2G), it still remained comparable activity of eGFP inhibition with OMeRNA-6PS across all the concentrations tested intracellularly.

At the concentration of 320 nM, FRNA-6PS, OMeRNA-6PS, and MOERNA-6PS all exhibited significant reductions in both fluorescent signals and eGFP mRNA copy numbers within a recorded time window of 72 h post-transfection (Fig. 4D and E and Supplementary Fig. S7B). Importantly, ∼90% of eGFP expression was inhibited 24 h post-transfection, with minimal discernible differences among the three constructs. The inhibition reached its maximum at 48 h post-transfection. Remarkably, even at the 72-h mark, when cell division had led to a substantial decrease in the effective intracellular oligonucleotide concentrations, >50% reduction in eGFP expression was still achieved, even with the least active FRNA-6PS. This reduction, although somewhat decreased compared with the earlier timepoints, underscores the persistent potency of FRNA-6PS, OMeRNA-6PS, and MOERNA-6PS. Given the robustness of the CMV promoter, it was plausible to reason that each catalytic molecule have targeted multiple copies of mRNA within the cells to realize such robust gene-silencing effects under continuous eGFP expression conditions. The time-dependent inhibition patterns further reinforce the notion that the catalytic efficiency of these molecules might be modulated by interactions with intracellular proteins as their concentrations decrease.

### Detection of mRNA cleavage products by FRNA-6PS, OMeRNA-6PS, or MOERNA-6PS in HeLa cells via RACE PCR and Sanger sequencing

To further confirm that the observed eGFP mRNA knockdown was due to the site-specific cleavage mechanism via the intrinsic catalytic activity of ASO-like variants, we directly detected the eGFP mRNA 3′ cleavage products in HeLa cells using 5′ RACE PCR (Supplementary Fig. S8A). RACE amplicons of the expected molecular weight derived from ASO-like 10–23 variants-treated HeLa cells were observed by agarose gel electrophoresis, corresponding to the successful reverse transcription and amplification of both the uncleaved and the catalytically cleaved mRNA of eGFP (Supplementary Fig. S8B). Sanger sequencing of the RACE amplicons derived from the cleaved mRNA (Supplementary Fig. S8C) revealed that most of the amplicons contained the 5′ terminus of eGFP mRNA with the expected post-cleavage site sequence (Fig. 4F and Supplementary Fig. S8D). A few RACE PCR amplicons that contained 5′ terminus of eGFP mRNA missing a few nucleotides adjacent to the cleavage site and locating in the enzyme-binding arm region were only observed for samples derived from HeLa cells treated by MOERNA-6PS, corresponding to putative RNaseH1-mediated cleavage sites (Supplementary Fig. S8D). This was consistent with our *in vitro* observation that MOERNA-6PS triggered more RNase H-mediated RNA degradation relative to FRNA-6PS and OMeRNA-6PS, likely due to the higher composition of DNA in the substrate-binding arms in close proximation to the cleavage site (Supplementary Fig. S4).

### Intracellular inhibition of endogenously expressed c-MYC oncogene in colon cancer HCT116 cells

**Figure 5.**
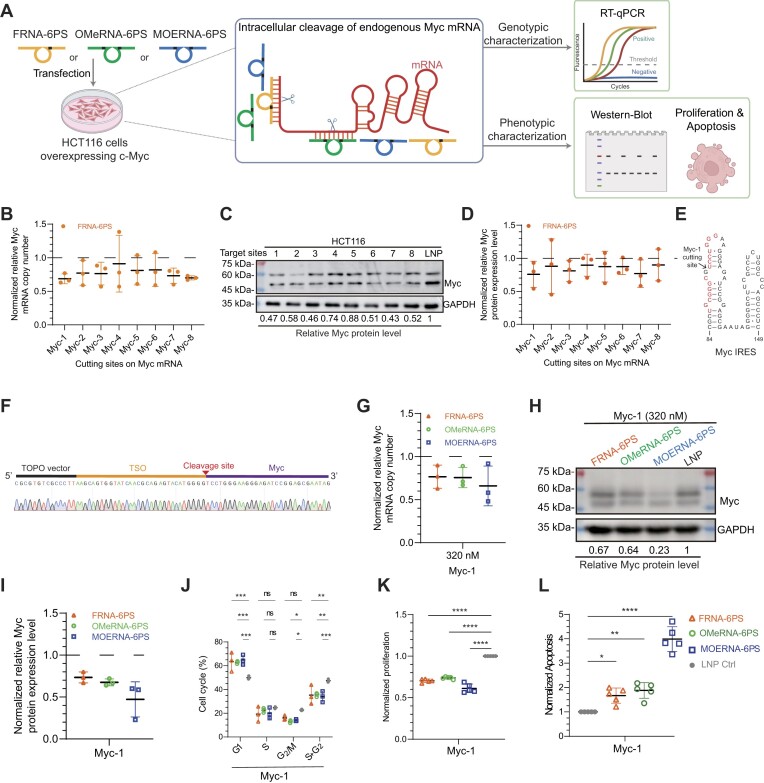
Intracellular inhibition of endogenously over-expressed c-MYC oncogene in colon cancer HCT116 cells. (**A**) Schematic representation of using FRNA-6PS, OMeRNA-6PS, and MOERNA-6PS molecules for the intracellular inhibition of endogenously over-expressed oncogenic c-MYC. Genotypically, RT-qPCR was performed to quantitatively assess the catalytic reduction of Myc mRNA copy number. Phenotypically, the effects on the level of Myc protein expression, cell cycle, proliferation, and apoptosis were quantitatively assessed. (B–D) The effect of FRNA-6PS designed to target different sites on Myc RNA. (**B**) RT-qPCR analysis of DNA-free total RNA isolated from HCT116 cells 24 h post-transfection of FRNA-6PS variants using Myc-specific and GAPDH loading control primers. The mRNA copy numbers of c-MYC were normalized to that of GAPDH. (**C**) Representative SDS–PAGE showing the western blot of c-Myc protein derived from HCT116 cells 24 h post-transfection of FRNA-6PS variants. (**D**) Analysis of c-Myc protein expression levels in HCT116 cells 24 h post-transfection of FRNA-6PS variants by western blot. The c-Myc protein expression levels for cells transfected by FRNA-6PS variants were normalized to that for cells transfected with PBS control [denoted as LNP in panel (C)]. Error bars are ±s.d. of the mean for *n* = 3 technical replicates in panels (B) and (D). (**E**) The thermodynamically stable secondary structure of c-MYC IRES comprising two hairpins and three internal loops. The Myc-1 cutting site within this structure is indicated by the arrow. (**F**) 5′ RACE PCR and Sanger sequencing revealed the sequence derived from the 3′ cleavage product of FRNA-6PS mediated cleavage at the designed site (cleavage site, indicated by the arrow) of c-MYC IRES mRNA (MYC), appending to a RACE TSO. (G–I) The effect of ASO-like variants designed to target c-MYC IRES (Myc-1) to mRNA copy and protein expression. (**G**) RT-qPCR analysis of DNA-free total RNA isolated from HCT116 cells 24 h post-transfection of the three variants using c-MYC-specific and GAPDH loading control primers. The mRNA copy numbers of c-MYC were normalized to that of GAPDH. (**H**) Analysis of c-Myc protein expression levels in HCT116 cells 24 h post-transfection of the three variants by western blot. The c-Myc protein expression levels for cells transfected by ASO-like variants were normalized to that for cells transfected with PBS control [denoted as LNP in panel (H)]. Error bars are ±s.d. of the mean for *n* = 3 technical replicates in panels (G) and (H). (**I**) Representative SDS–PAGE showing the western blot of c-Myc protein derived from HCT116 cells 24 h post-transfection of the three variants. The effect of FRNA-6PS, OMeRNA-6PS, and MOERNA-6PS targeting c-MYC IRES (Myc-1) on cell cycle (**J**), proliferation (**K**), and apoptosis (**L**). Three biological replicates and three (panel J) or five (panels K and L) technical replicates were collected, with one representative biological replicate shown. Error bars are ±s.d. of the mean for *n* = 3 or 5 technical replicates (*P < .05; **P < .01; ***P < .001; ****P < .0001; ns, not significant).

c-MYC is an oncogenic transcription factor that is often constitutively expressed in many cancer types and coordinately regulates a network of proteins involved in cell cycle, proliferation, and metabolism [38, 39]. Target-specific inhibition of c-MYC could represent a general therapeutic strategy for a broad spectrum of cancers. However, c-Myc has been considered difficult-to-drug at the protein level due to the lack of well-defined ligand-binding pocket [40, 41], which renders targeted inhibition of c-MYC mRNA an attractive approach [42, 43]. Therefore, having established that all the three ASO-like 10–23 variants can achieve potent and durable inhibition of constitutively expressed exogenous eGFP in HeLa cells, we further established their activities on endogenous c-MYC mRNA knockdown in colon cancer HCT116 cells (Fig. 5A).

We initially screened cleavage sites on c-MYC mRNA within the internal ribosome entry site (IRES) and protein-coding regions (Supplementary Fig. S9A) to identify optimal sites for ASO-like 10–23 enzyme activity. Using the intracellularly least active FRNA-6PS as the probing molecule, we designed eight FRNA-6PS variants targeting different rG–rU junctions located in distinct context of secondary structures (Supplementary Fig. S9B). When HCT116 cells were transfected with 320 nM of these variants, c-MYC RNA levels decreased to ∼70%–90% after 24 h (Fig. 5B), accompanied by a reduction in c-Myc protein to ∼75%–90% (Fig. 5C and D). Similar to ASOs, the silencing efficiency of ASO-like DNAzymes was highly dependent on the structural and sequence context of the target RNA, underscoring the importance of target site screening through strategies such as “ASO walking” [22].

Among the eight cutting sites, Myc-1, located in the thermodynamically stable secondary structure of the c-MYC IRES, which includes two hairpins and three internal loops (Fig. 5E), was selected for further study due to its poor accessibility by conventional oligonucleotide-based approaches [44]. FRNA-6PS targeting Myc-1 exhibited the most efficient reduction in both mRNA levels and protein expression among the eight selected cutting sites (Fig. 5B–D), which suggested c-MYC IRES as an appropriate silencing target by our ASO-like catalytic molecules. 5′ RACE PCR and Sanger sequencing confirmed cleavage at the expected Myc-1 cutting site (Fig. 5F and Supplementary Fig. S10), validating the mechanism of c-MYC mRNA inhibition through catalytic cleavage.

Not surprisingly, when HCT116 cells were transfected with OMeRNA-6PS or MOERNA-6PS (both at 320 nM) targeting Myc-1, both mRNA and protein levels were further reduced compared with FRNA-6PS after 24 h (Fig. 5G–I), with MOERNA-6PS achieving the most pronounced reduction in c-Myc protein to ∼50% (Fig. 5H and I). Notably, compared with a potent Myc-RIBOTAC strategy that also targets the Myc IRES for degradation, all three ASO-like 10–23 variants achieved greater reductions in both c-MYC mRNA and protein expression. These reductions were associated with G1 phase cell cycle arrest (Fig. 5J), decreased proliferation (Fig. 5K), and increased apoptosis (Fig. 5L) in HCT116 cells.

## Discussion

Chemical modifications play a crucial role in enhancing the stability, efficacy, and metabolic properties of nucleic acid therapeutics. Among these modifications, RNA analogues such as FRNA, OMeRNA, and MOERNA, along with backbone modifications like PS and phosphorodiamidate morpholino (PMO) [45], have contributed to the development of approved ASO and siRNA drugs across generations of modifications [5, 6, 24]. These modifications serve multiple purposes, including bolstering resistance against nucleolytic degradation. Specifically, FRNA, OMeRNA, and MOERNA are strategically applied to the seed regions of ASOs or selectively integrated within siRNA sequences to enhance the recognition of RNA targets. Partial or complete substitution of PS and PMO internucleotide linkages further enhances the drugs’ metabolic stability. The molecular patterns observed in these well-established drugs serve as immediate and pertinent references for the development of next-generation oligonucleotide therapeutics.

DNAzyme 10–23 stands out as a promising oligonucleotide drug candidate, uniquely capable of achieving allele-specific RNA silencing beyond the capabilities of traditional ASO or siRNA counterparts. Through strategic chemical modifications, various variants of DNAzyme 10–23 have been engineered to exhibit enhanced biological stability and catalytic efficiency under physiologically compatible conditions or within cellular environments. Notable examples include X10–23 and Dz 46, which have harnessed comprehensive modifications derived from the advancements in XNA chemistry. Nonetheless, the potency and toxicity of oligonucleotide drugs are profoundly influenced by their molecular configurations. Previous studies have demonstrated that introducing a single 2′-OMe modification at gap position 2 within ASO molecules generally diminishes protein binding, leading to a significant reduction in hepatotoxicity and an overall improvement in the therapeutic index [29]. Given the intricate spectrum of molecular variants encompassed by a single 10–23 DNAzyme variant, it remains crucial to comprehensively assess the extent to which such modified 10–23 constructs can achieve the requisite activity, safety, and optimal pharmacokinetic and pharmacodynamic properties essential for clinical applications.

In this study, we have undertaken an innovative approach of grafting the molecular patterns and chemical constituents found in approved ASO drugs into the sequence of 10–23, with the goal of maximizing the emulation of ASO attributes within the framework of 10–23. In the design of FRNA-6PS, OMeRNA-6PS, and MOERNA-6PS, we introduced RNA binding-enhancing RNA analogues into the substrate-binding arms, mimicking the seed regions of ASOs. This strategic modification facilitated RNA substrate association even within the context of complex secondary and tertiary RNA structures, and achieved enhanced turnover capability through a good balance between substrate association pre-catalysis and cleavage product release post-catalysis. Furthermore, several discrete PS internucleotide linkages in the catalytic core should not only reduce the formation of catalytically inactive homoduplexes induced by palindromic sequences, but also might function at least in some extent to promote interactions with plasma and cytosolic proteins, like their function in ASOs. Gratifyingly, FRNA-6PS, OMeRNA-6PS, and MOERNA-6PS demonstrated significantly enhanced turnover capabilities compared with the WT, especially when enzymes were present at substoichiometric concentrations. These enhanced activities were further amplified when these modified DNAzymes were used to cleave a full-length mRNA sequence spanning 736 nt encoding eGFP under simulated physiological conditions. This achievement meets several key prerequisites for potential therapeutic applications. We anticipate that our design principle would be sufficiently versatile to accommodate expanding chemical modifications that have been identified as critical to the success of ASO and siRNA drugs. This approach can be applied not only to the DNAzyme 10–23 but also to other DNAzymes, particularly DNAzyme 8–17, the structure of which has already been determined [46].

The elimination of RNase H-mediated substrate cleavage, as observed with FRNA-6PS, and the substantial suppression witnessed with OMeRNA-6PS and MOERNA-6PS, stand as notable achievements. These RNA analogue modifications to the substrate-binding arms exhibit a compelling dual effect: not only do they bolster intrinsic catalytic performance, characterized by enhanced turnover rates and improved accessibility to intricate RNA structures, but they also successfully counteract RNase H-mediated cleavage without sequence specificity. Our verification using RACE PCR coupled with Sanger sequencing of the eGFP and c-MYC target confirms the high accuracy of cleavage site specificity. RNA-cleaving nucleic acid enzymes have shown remarkable potential for allele-specific inhibition of disease-causing mRNA alleles that differ by a single nucleotide from their wild-type counterparts, an accuracy that exceeds that of traditional ASO and siRNA. However, “off-target” often associated with antisense mechanisms often comprises such an accuracy. By suppressing the RNase H-mediated antisense pathway, these ASO-like 10–23 variants present a promising therapeutic approach to mitigate the risk of off-target effects. This advancement highlights their potential in selectively targeting disease-associated single-nucleotide polymorphisms, a focus of ongoing studies in our laboratory.

Within the cell, we anticipated that the therapeutic effectiveness of FRNA-6PS, OMeRNA-6PS, and MOERNA-6PS, akin to PS-ASO drugs, would intertwin with their interactions with cellular proteins. These interactions, in turn, could impact their subcellular localization and their ability to engage with RNA substrates for catalysis. Our investigations have now confirmed our expectations, revealing that these oligonucleotides indeed engage in protein interactions of varying degrees. Importantly, we observed that lower administration dosages through transfection led to more pronounced interference from such interactions. Notably, among the three variants, FRNA-6PS exhibited the greatest level of binding to cytosolic proteins. Consequently, the intracellular eGFP inhibition activity of FRNA-6PS did not consistently follow a dose-dependent pattern when HeLa cells were transfected with <160 nM of FRNA-6PS. Importantly, our studies indicated no cytotoxicity when cells were transfected with doses of up to 500 nM for any of the three variants. We posit that the binding of FRNA-6PS, OMeRNA-6PS, and MOERNA-6PS to cytosolic protein may act as natural reservoirs, shielding these molecules from rapid degradation or excretion by cells.

One notable advantage of oligonucleotide drugs lies in their ease of conjugation with other functional modalities. This can be achieved by appending chemical reactive groups to the 5′ and 3′ termini or internal nucleobases without compromising the biological activity of the oligomers themselves. Harnessing this property, it is clear that via proper molecular conjugation to functional modalities that direct subcellular localization at target rich organelles, such as nuclear localization signal peptide for enhanced nuclear entry, along with the screening of efficient delivery systems, exceptional potency, as well as favorable *in vivo* pharmacokinetic and pharmacodynamic properties can also be achieved by these molecules. Investigations addressing these aspects are currently ongoing in our laboratory.

In summary, we have engineered 10–23 variants inspired by ASOs, resulting in improved RNA cleavage efficiency *in vitro* and enhanced mRNA-silencing potency in cells. Leveraging the expanding array of chemistries applied in successful oligonucleotide therapeutics, the ongoing refinement of classical nucleic acid enzymes through strategic molecular design holds promising prospects. These ASO-like RNA-cleaving nucleic acid enzymes, fortified by deliberate chemical modifications, represent a significant step toward therapeutic DNAzyme development, and offer exciting potential to complement gene-silencing therapeutics with substrate selectivity of single-nucleotide precision.

## Supplementary Material

gkaf144_Supplemental_File

## Data Availability

All data are available from the authors upon request.
