# Supplementary material for: Chemical evolution of ASO-like DNAzymes for effective and extended gene silencing in cells
Source: Nucleic Acids Res. 2025 Mar 4;53(5):gkaf144. doi: 10.1093/nar/gkaf144 (PMC11879453; doi:10.1093/nar/gkaf144)
Supplement: gkaf144_Supplemental_File [file gkaf144_supplemental_file.docx]

**Supplementary Information**

**Chemical Evolution of ASO-Like DNAzymes for Effective and Extended Gene Silencing in Cells**

Yingyu Liu^1^, Sheyu Zhang^1^, Meiqi Zhang^1,3^, Xin Liu^1,4^, Yashu Wu^1^, Qin Wu^1, 2^, John C. Chaput^5, 6, 7, 8^, Yajun Wang^1, 2*^

^1^Hangzhou Institute of Medicine (HIM), Chinese Academy of Sciences, Hangzhou, Zhejiang 310000, China.

^2^The Cancer Hospital of the University of Chinese Academy of Sciences (Zhejiang Cancer Hospital), Hangzhou, Zhejiang 310022, China

^3^Hangzhou Institute for Advanced Study, University of Chinese Academy of Sciences, Hangzhou, Zhejiang 310024, China

^4^Zhejiang University of Technology, College of Pharmaceutical Science, Hangzhou, Zhejiang 310014, CN

^5^Department of Pharmaceutical Sciences, University of California, Irvine, CA 92697-3958, USA

^6^Department of Chemistry, University of California, Irvine, CA 92697-3958, USA.

^7^Department of Molecular Biology and Biochemistry, University of California, Irvine, CA 92697-3958, USA

^8^Department of Chemical and Biomolecular Engineering, University of California, Irvine, CA 92697-3958, USA

^*^ To whom correspondence should be addressed to: wangyajun@him.cas.cn

**TABLE OF CONTENTS**

**1. Supplementary Figures**

Figure S1. Thermal melting study of homoduplex formed by 10-23 containing wild-type or multiple PS substituted catalytic cores

Figure S2. Sequence scheme of enzyme-substrate complex used in the modification pattern optimization of ASO-like 10-23

Figure S3. Pseudo first-order catalytic activity of ASO-like 10-23 targeting different sites of eGFP mRNA

Figure S4. Analysis of the RNA cleavage mediated by the intrinsic catalytic activity of ASO-like 10-23s and/or RNase H activity.

Figure S5. The intracellular stability of FRNA-6PS, OMeRNA-6PS, and MOERNA-6PS

Figure S6. The simulated intracellular stability of FRNA-6PS, OMeRNA-6PS, and MOERNA-6PS

Figure S7. Bright field images of cells in Fig 4b and 4d

Figure S8. Detection of FRNA-6PS, OMeRNA-6PS, or MOERNA-6PS cleavage products in HeLa cells by RACE PCR and Sanger sequencing

Figure S9. Design of FRNA-6PS, OMeRNA-6PS, or MOERNA-6PS to cleave different sites of c-Myc mRNA

Figure S10. Detection of FRNA-6PS cleavage products of Myc-1 mRNA in HCT116 cells by RACE PCR and Sanger sequencing

**2. Table of oligonucleotides**

Table S1. Table of all oligonucleotides

Table S2. Table of primers used for qPCR

Table S3. Table of oligos and primers used for 5’RACE PCR

**Supplementary Figures**

**Figure S1**

**
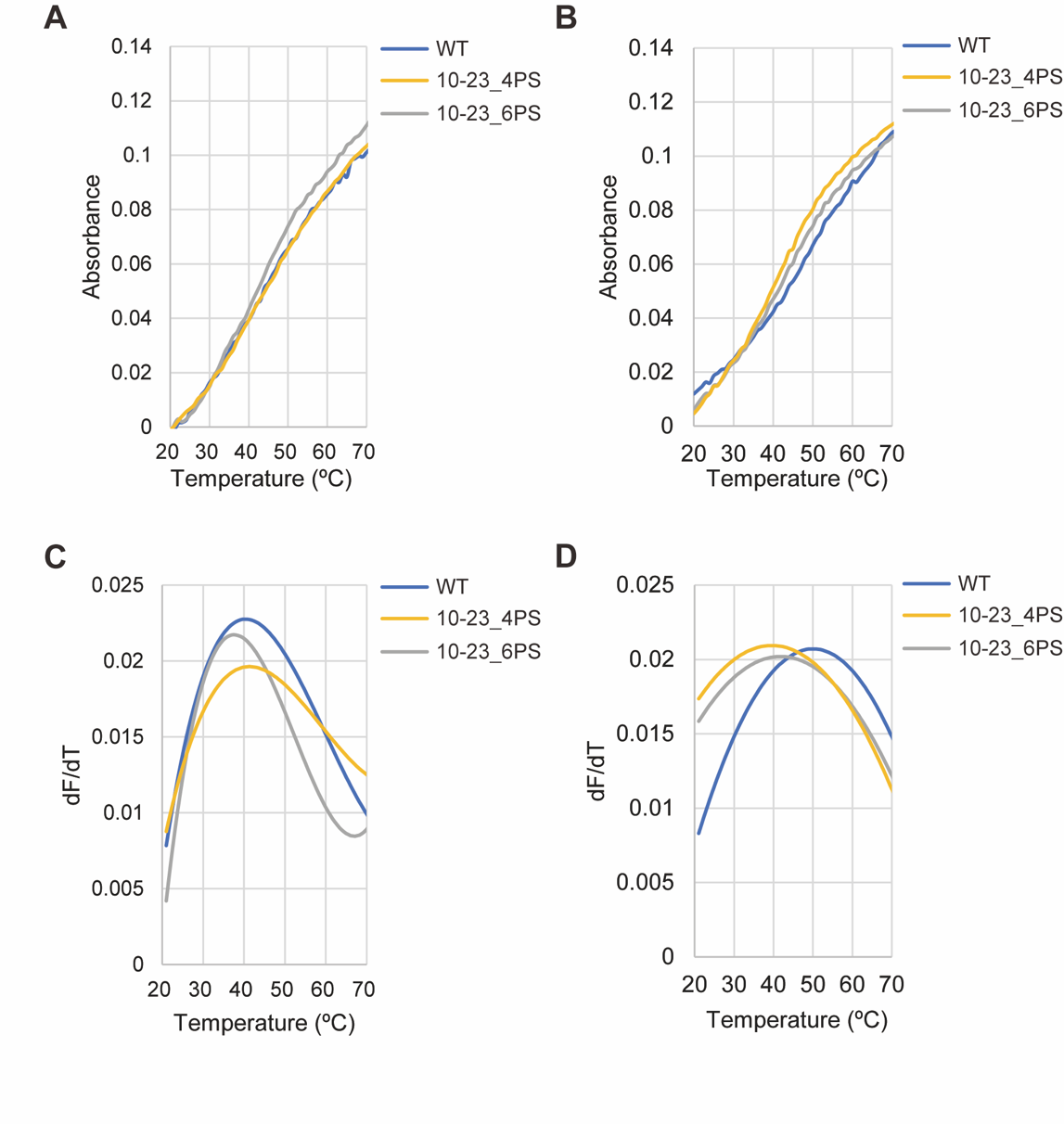
**

**Figure S1.** Thermal melting study of homoduplex formed by 10-23 WT, 10-23_4PS, and 10-23_6PS. All thermal melting measurements were performed in 50 mM Tris-HCl (pH 7.5) buffer containing 2.5 mM MgCl_2_, 200 mM NaCl, with each variant version of 10-23 poised at 3 µM. (A)-(B)The average absorbance of three separate forward (A) and reverse (B) measurements at 260 nm over a temperature range of 20 to 85°C. (C)-(D) Plots of the first derivative dF/dT vs. temperature of forward (C) and reverse (D) measurements.

**Figure S2**

**
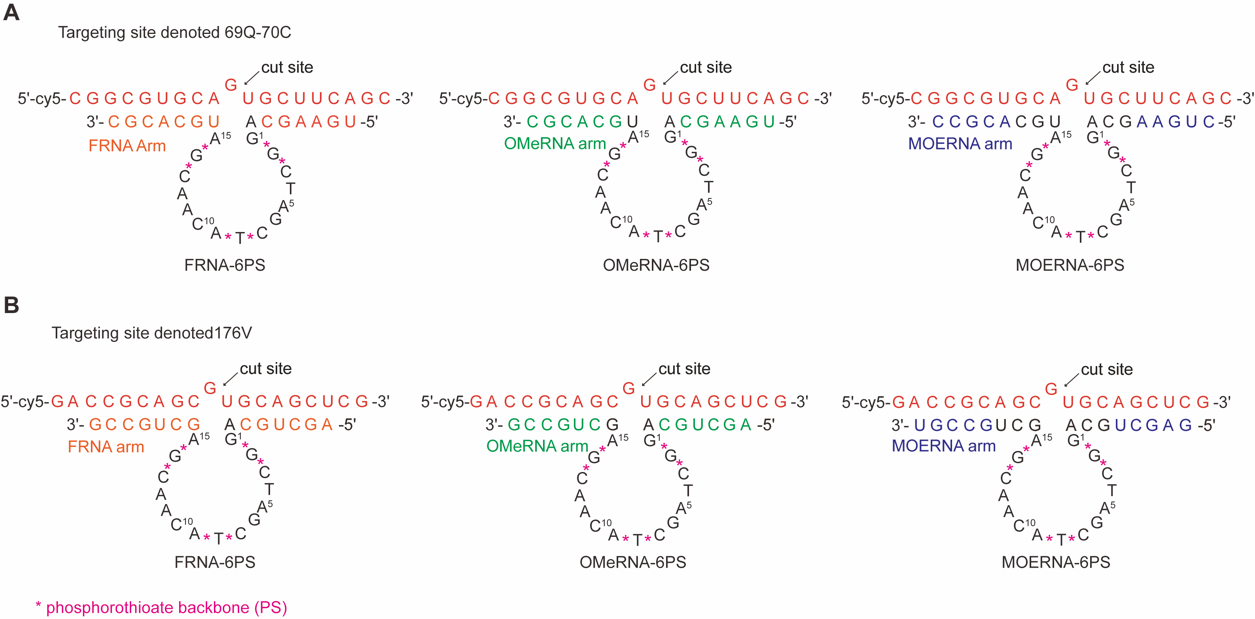
**

**Figure S2.** Sequence scheme of enzyme-substrate complex used in the modification pattern optimization of ASO-like 10-23. 5’ Cy5 labeled RNA substrate (top strand, 5’ to 3’) derived from mRNA of eGFP denoted targeting site 69Q-70C (A) and (B) 176V in complex with 10-23 of different modification patterns (bottom strand, 3’ to 5’). FRNA residues are in orange, OMeRNA residues are in green, MOERNA residues are in blue, and DNA residues are in black. Asterisks in magenta denote backbone phosphorothioate linkage (PS) in the catalytic core. The arrow indicates the catalytic cleavage site.

**Figure S3**

**
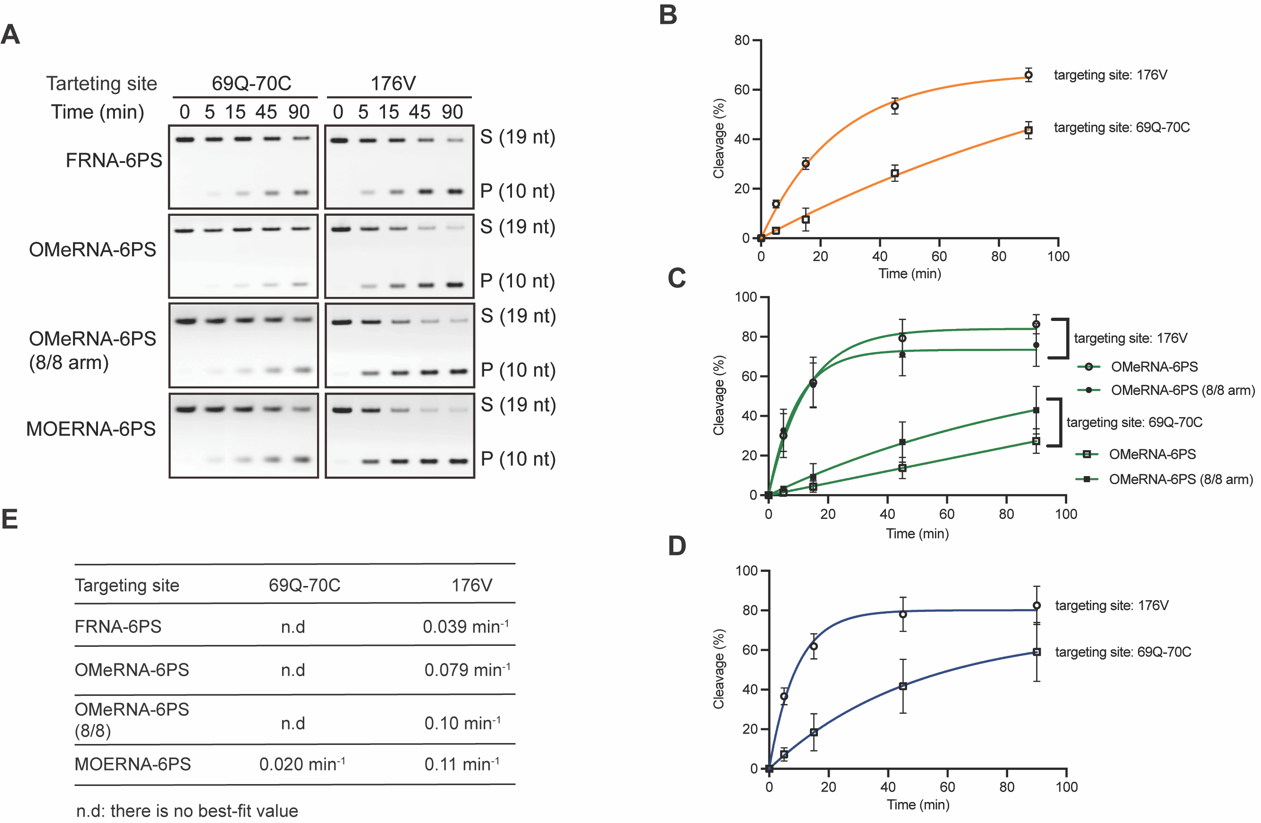
**

**Figure S3.** Pseudo first-order catalytic activity of ASO-like 10-23 targeting different sites of eGFP mRNA. (A) Representative PAGE gels showing the single-turnover catalytic activity of ASO-like 10-23 of different configurations cleaving 5’ Cy5 labeled 19 nt RNA substrates derived from eGFP and denoted 69Q-70C and 176V, respectively. S, substrate; P, cleavage product. (B-D) Pre-steady-state kinetic analysis of RNA cleavage by FRNA-6PS (B) OMeRNA-6PS of different substrate binding arm length (C) and MOERNA (D). Error bars denote ± s.d. of the mean for n = 3 independent replicates. All cleavage reactions were performed under single-turnover conditions with 0.3 μM substrate and 1.5 μM enzyme in 50 mM Tris-HCl (pH 7.5) containing 1 mM MgCl_2_ and 200 mM NaCl at 37°C. E, Summary of first-order rate constants (*k*_obs_) depicted by the kinetic plots in B-D.

**Figure S4**

**
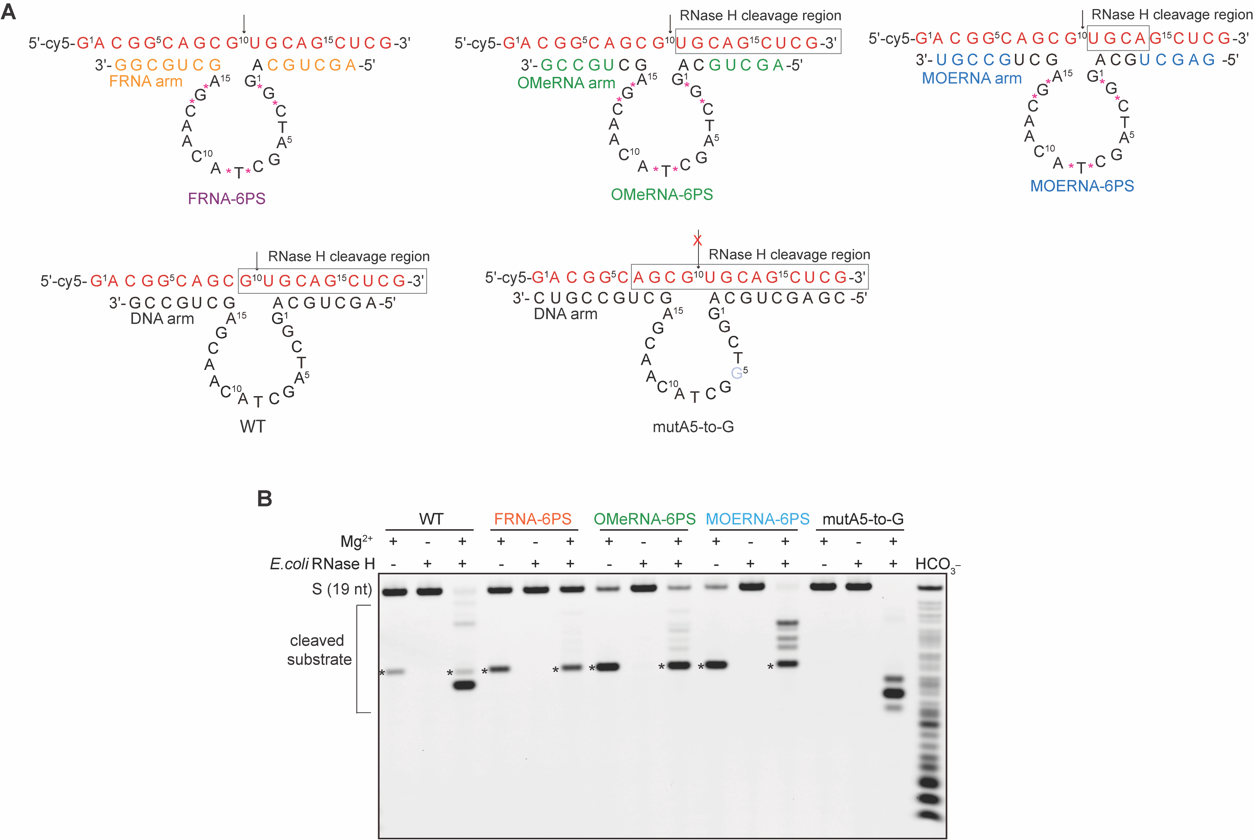
**

**Figure S4.** Analysis of the RNA cleavage mediated by the intrinsic catalytic activity of ASO-like 10-23s and/or RNase H activity. (A) Constructs used in the analysis. 5’ Cy5 labeled RNA substrate (top strand, 5’ to 3’) in complex with 10-23 of different configurations (bottom strand, 3’ to 5’). FRNA residues are in orange, OMeRNA residues are in green, MOERNA residues are in blue, and DNA residues are in black. Asterisks in magenta denote backbone PS in the catalytic core. The arrow indicates the catalytic cleavage site of 10-23 at the ribo-G_10_. The boxed region indicates RNase H mediated non-site specific cleavage in each case of ASO-like modification pattern, among which FRNA-6PS displays autonomous catalytic cleavage activity without evoking RNase H mediated RNA degradation. (B) Analysis of the RNA cleavage mediated by the intrinsic catalytic activity of ASO-like 10-23s (asterisks marked bands) and/or RNase H activity resolved by representative PAGE gel. All cleavage reactions were performed under single-turnover conditions with 0.3 μM substrate and 1.5 μM enzyme in 50 mM Tris-HCl (pH 7.5) containing 1 mM MgCl_2_ and 200 mM NaCl for 20 min at 37°C.

**Figure S5**

**
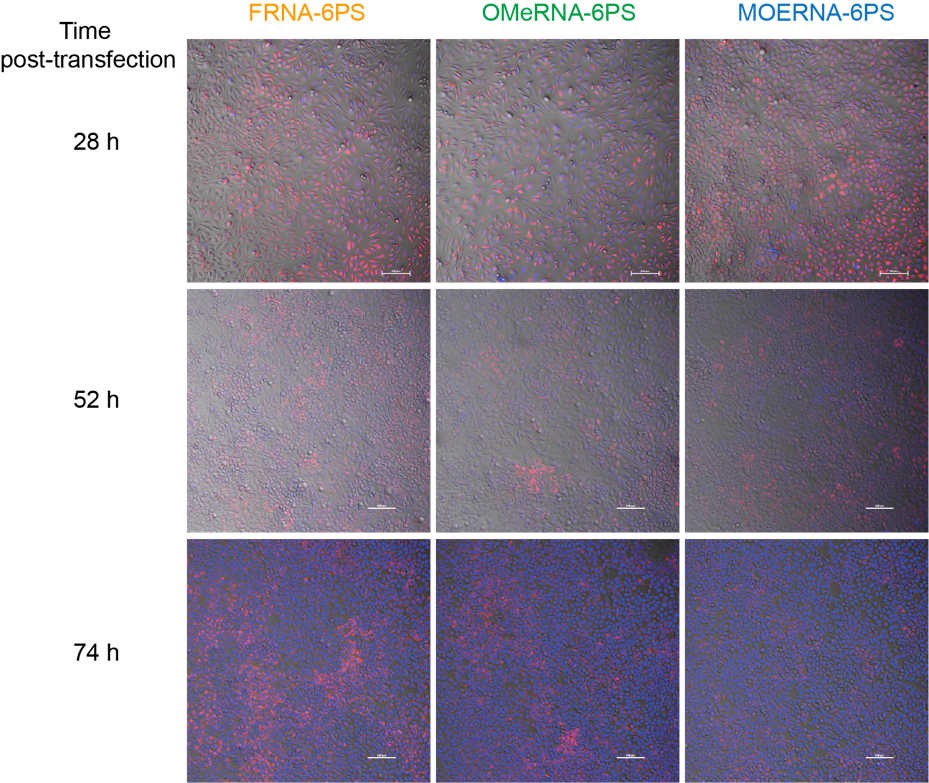
**

**Figure S5.** The intracellular stability of FRNA-6PS, OMeRNA-6PS, and MOERNA-6PS. Confocal images of HeLa cells 28, 52, and 74 hours after transfected with 160 nM of 5’-Cy5 dye labeled FRNA-6PS, OMeRNA-6PS, or MOERNA-6PS (shown in red). All the three types of molecules displayed long-lasting existence in cells. Hoechst 33258 was used to label nucleus (blue). Scale bar, 100 µm.

**Figure S6**

**
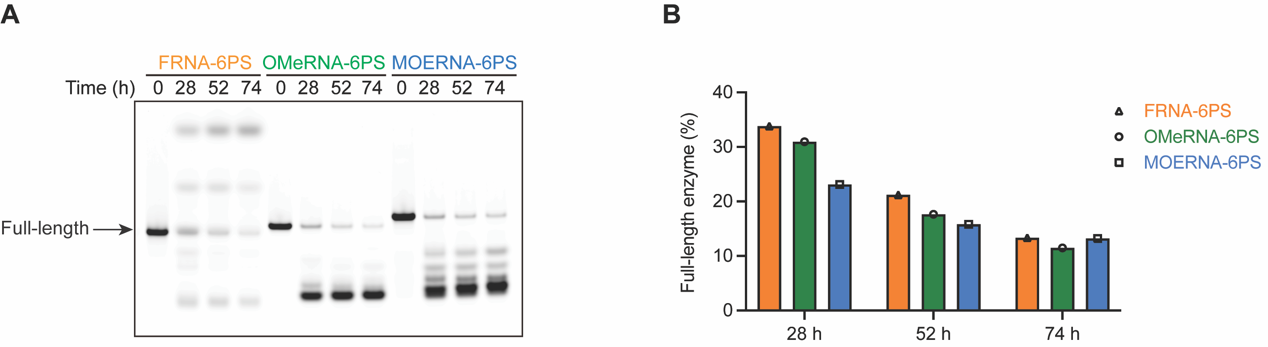
**

**Figure S6.** The simulated intracellular stability of FRNA-6PS, OMeRNA-6PS, and MOERNA-6PS. (A) Representative PAGE analysis of FRNA-6PS, OMeRNA-6PS, and MOERNA-6PS upon incubation in whole cell lysate for 28, 52, and 74 hours at 37°C. (B) The quantitatively assessed percentage of full-length FRNA-6PS, OMeRNA-6PS, or MOERNA-6PS at each time point as shown in (A).

**Figure S7**

**
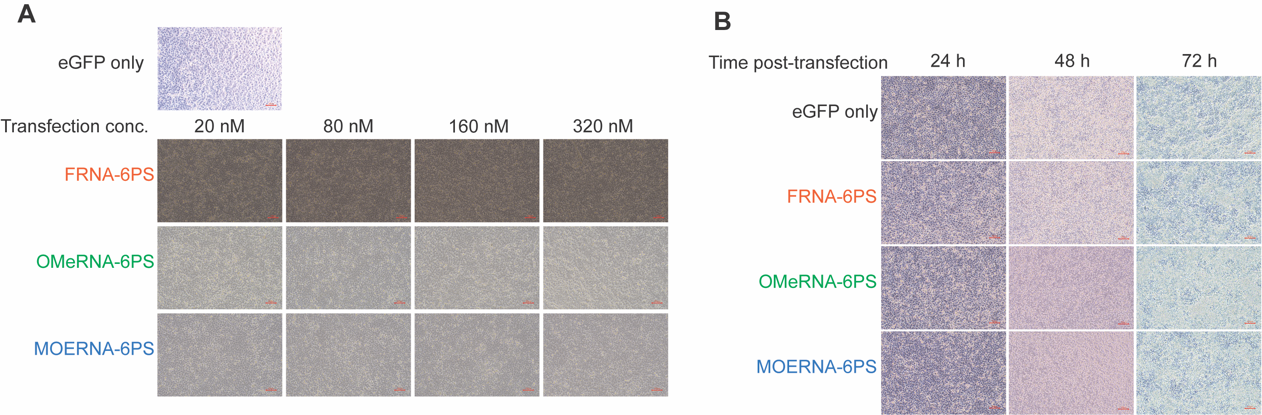
**

**Figure S7.** Bright field images of cells for dose-dependent eGFP inhibition in Figure 4B (A), and time-dependent eGFP inhibition in Figure 4D (B). Scale bar, 100 µm.

**Figure S8**

**
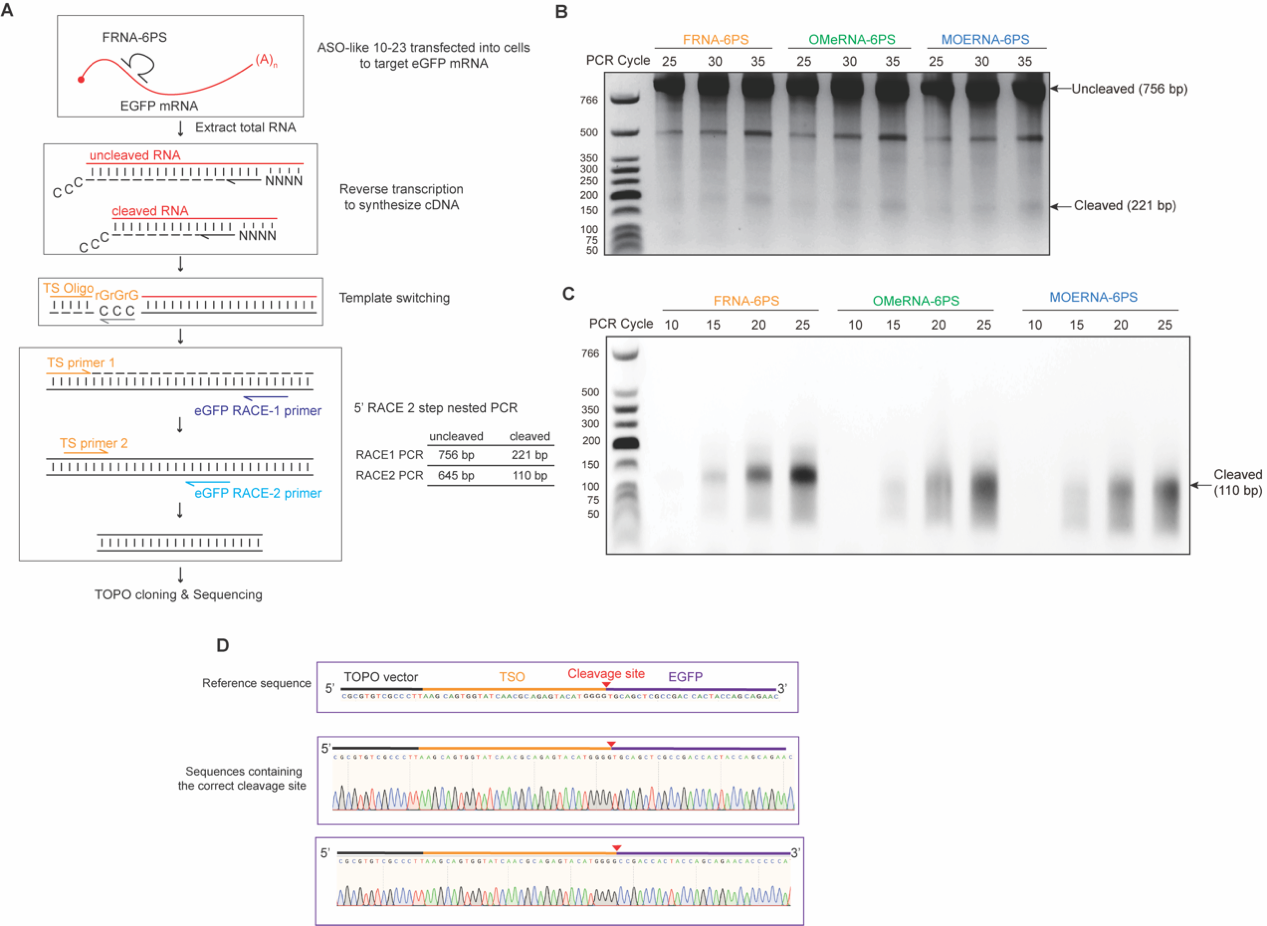
**

**Figure S8.** Detection of mRNA cleavage products by FRNA-6PS, OMeRNA-6PS, or MOERNA-6PS in HeLa cells via RACE PCR and Sanger sequencing. (A) Schematic outline of the experimental protocol for the detection of intracellular cleavage product of eGFP catalyzed by ASO-like 10-23 variants transfected to HeLa cells through 5’ RACE PCR followed by TOPO cloning and Sanger sequencing. (B and C) Representative agarose gel electrophoresis showing RACE1 (B) and RACE2 (C) PCR amplicons of the uncleavage and cleavage products. (D) 5’ RACE PCR and Sanger sequencing revealed the sequence derived from the 3’ cleavage product of ASO-like 10-23 variants mediated cleavage (cleavage site, indicated by the red arrow) of eGFP mRNA (eGFP, indicated in purple), appending to a RACE template-switching oligo (TS, indicated in orange). Few RACE PCR amplicons that contained 5′ terminus of eGFP mRNA missing a few nucleotides adjacent to the cleavage site and locating in the enzyme binding arm region were only observed for samples derived from HeLa cells treated by MOERNA-6PS, corresponding to putative RNaseH1-mediated cleavage sites (bottom panel)**.**

**Figure S9**

**
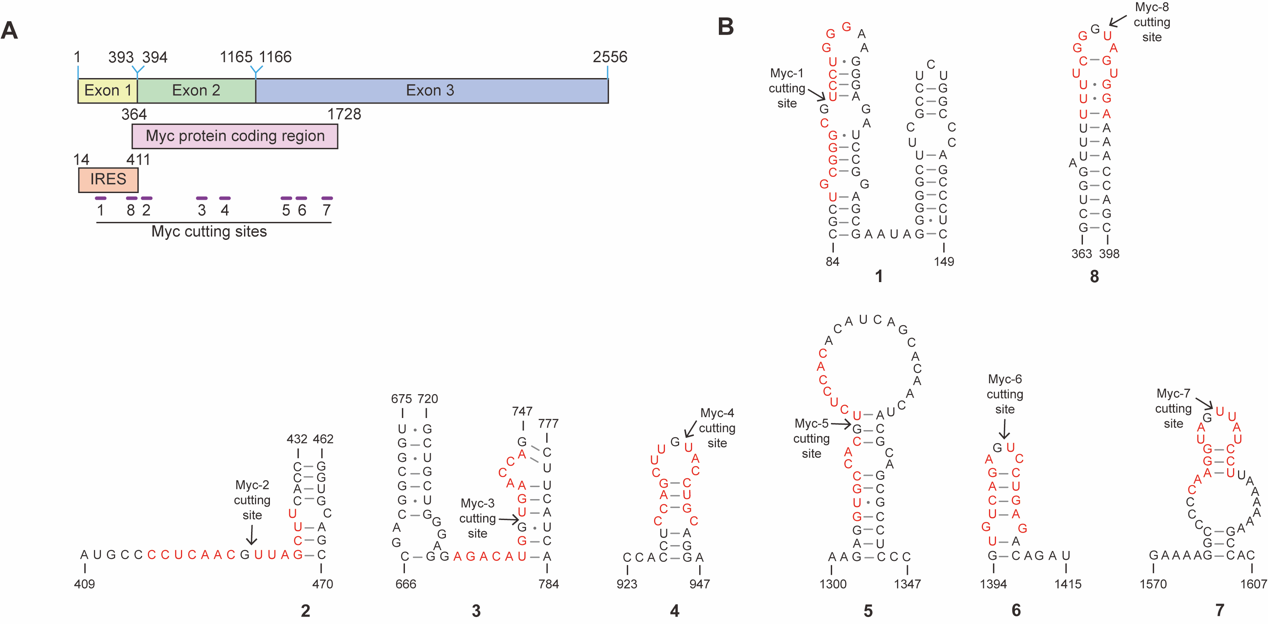
**

**Figure S9.** Design of FRNA-6PS, OMeRNA-6PS, or MOERNA-6PS to cleave different sites of c-MYC mRNA. (A) Schematic representation of the frame of c-MYC mRNA and the location of the eight designed cutting sites. (B) The secondary structure and sequence context of the eight cutting sites (1-8) by ASO-like 10-23 molecules. The cutting sites are indicated by the black arrows.

**Figure S10**

**
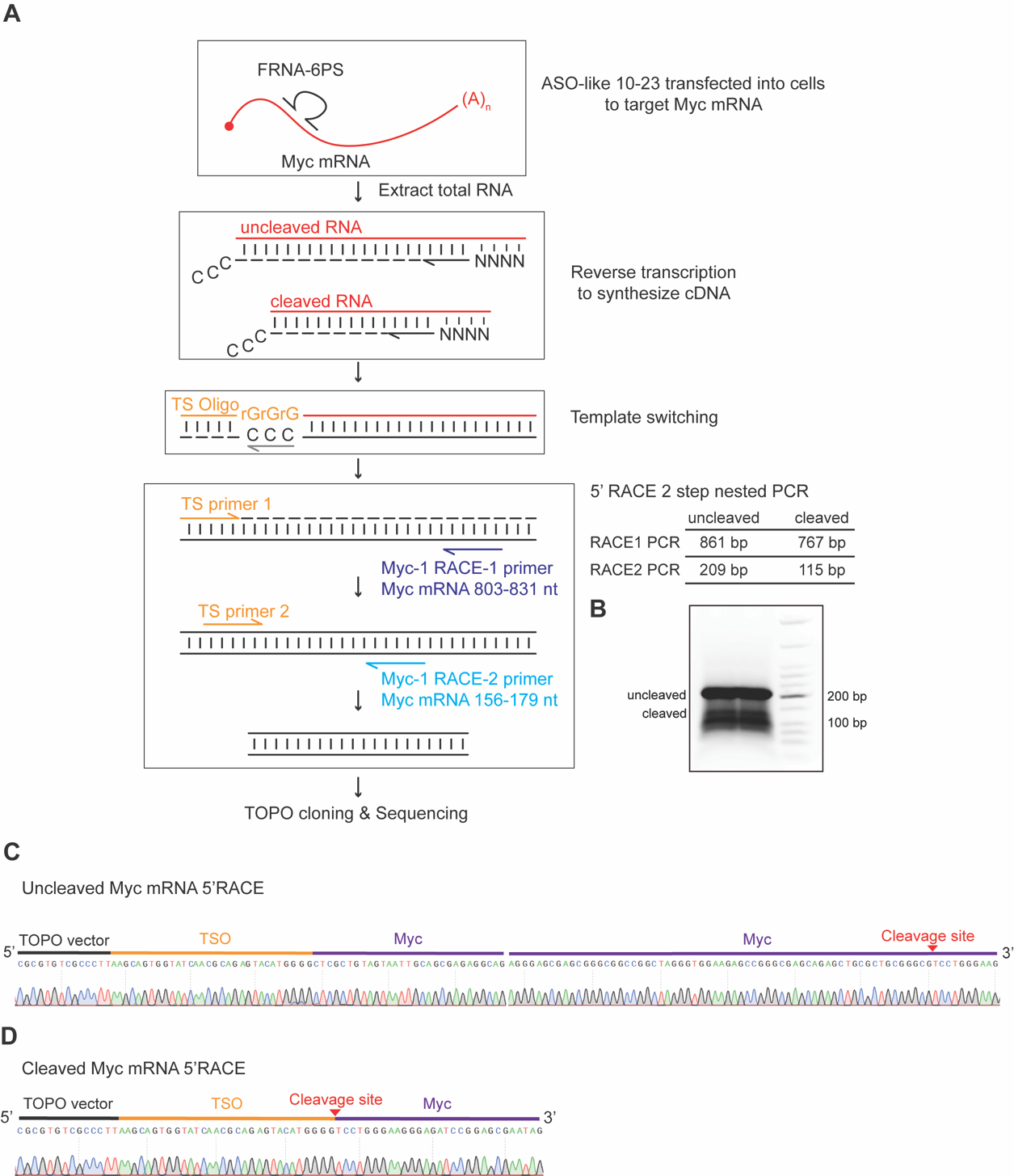
**

**Figure S10.** Detection of FRNA-6PS cleavage products of Myc-1 mRNA in HCT116 cells by RACE PCR and Sanger sequencing. (A) Scheme outline of the experimental protocol for the detection of intracellular cleavage product of c-MYC mRNA at cutting site Myc-1 catalyzed by FRNA-6PS transfected to HeLa cells through 5’ RACE PCR followed by TOPO cloning and Sanger sequencing. (B) Representative agarose gel electrophoresis showing the PCR amplicons of the uncleavage and cleavage products after RACE2 PCR. (C and D) 5’ RACE PCR and Sanger sequencing revealed the sequence derived from both the uncleaved and the 3’ cleavage product of FRNA-6PS mediated cleavage (cleavage site, indicated by the red arrow) of Myc mRNA (Myc, indicated in purple), appending to a RACE template-switching oligo (TSO, indicated in orange).

**Table of oligonucleotides**

**Table S1.** Table of all oligonucleotides

| **Oligo Name** | **Sequence (5' to 3')** |
| --- | --- |
| Oligonucleotides for mapping PS substitutions in the PO linker | |
| WT | CTACGCCAAGGCTAGCTACAACGAAGCTCCAAC |
| PS 1G | CTACGCCAA*GGCTAGCTACAACGAAGCTCCAAC |
| PS 2G | CTACGCCAAG*GCTAGCTACAACGAAGCTCCAAC |
| PS 3C | CTACGCCAAGG*CTAGCTACAACGAAGCTCCAAC |
| PS 4T | CTACGCCAAGGC*TAGCTACAACGAAGCTCCAAC |
| PS 5A | CTACGCCAAGGCT*AGCTACAACGAAGCTCCAAC |
| PS 6G | CTACGCCAAGGCTA*GCTACAACGAAGCTCCAAC |
| PS 7C | CTACGCCAAGGCTAG*CTACAACGAAGCTCCAAC |
| PS 8T | CTACGCCAAGGCTAGC*TACAACGAAGCTCCAAC |
| PS 9A | CTACGCCAAGGCTAGCT*ACAACGAAGCTCCAAC |
| PS 10C | CTACGCCAAGGCTAGCTA*CAACGAAGCTCCAAC |
| PS 11A | CTACGCCAAGGCTAGCTAC*AACGAAGCTCCAAC |
| PS 12A | CTACGCCAAGGCTAGCTACA*ACGAAGCTCCAAC |
| PS 13C | CTACGCCAAGGCTAGCTACAA*CGAAGCTCCAAC |
| PS 14G | CTACGCCAAGGCTAGCTACAAC*GAAGCTCCAAC |
| PS 15A | CTACGCCAAGGCTAGCTACAACG*AAGCTCCAAC |
| PS All | CTACGCCAA*G*G*C*T*A*G*C*T*A*C*A*A*C*G*AAGCTCCAAC |
| PS -5A | CTACGCCAA*G*G*C*TA*G*C*T*A*C*A*A*C*G*AAGCTCCAAC |
| PS odd-numbered | CTACGCCAA*GG*CT*AG*CT*AC*AA*CG*AAGCTCCAAC |
| PS even-numbered | CTACGCCAAG*GC*TA*GC*TA*CA*AC*GAAGCTCCAAC |
| PS 8T, 9A, 14G, 15A (4PS) | CTACGCCAAGGCTAGC*T*ACAAC*G*AAGCTCCAAC |
| PS -5A, 8T, 9A, 14G, 15A | CTACGCCAA*G*G*C*T*A*G*C*T*A*C*A*A*C*G*AAGCTCCAAC |
| PS 2G, 3C, 14G, 15A | CTACGCCAA*G*GCTAGCTACAAC*G*AAGCTCCAAC |
| PS 2G, 8T, 9A, 14G, 15A | CTACGCCAA*GGCTAGC*T*ACAAC*G*AAGCTCCAAC |
| PS 3C, 8T, 9A, 14G, 15A | CTACGCCAAG*GCTAGC*T*ACAAC*G*AAGCTCCAAC |
| PS 2G, 3C, 8T, 91, 14C, 15A (6PS) | CTACGCCAA*G*GCTAGC*T*ACAAC*G*AAGCTCCAAC |
| Mutant (3C-A, 5A-C) | CTACGCCAAGGaTcGCTACAACGAAGCTCCAAC |
| Oligonucleotides for eGFP 69Q-70C, 176V | |
| 69Q-70C 19 nt all RNA substrate | /56FAM/rCrGrGrCrGrUrGrCrArGrUrGrCrUrUrCrArGrC |
| 69Q-70C WT 7-7 idT (7 nt binding arm) | TGAAGCAGGCTAGCTACAACGATGCACGC/3idT/ |
| 69Q-70C 6/7FRNA-6PS 5Cy5 3idT (7 nt binding arm) | /5Cy5//i2FU//i2FG//i2FA//i2FA//i2FG//i2FC/AG*G*CTAGC*T*ACAAC*G*A/i2FU//i2FG//i2FC//i2FA//i2FC//i2FG//i2FC//3iT/ |
| 69Q-70C 6OMeRNA-6PS 5Cy5 3idT (7 nt binding arm) | /5Cy5//i2OMeU//i2OMeG//i2OMeA//i2OMeA//i2OMeG//i2OMeC/AG*G*CTAGC*T*ACAAC*G*AT/i2OMeG//i2OMeC//i2OMeA//i2OMeC//i2OMeG//i2OMeC//3idT/ |
| 69Q-70C 6OMeRNA-6PS 5Cy5 3idT (8 nt binding arm) | /5Cy5//i2OMeC//i2OMeU//i2OMeG//i2OMeA//i2OMeA//i2OMeG/CAG*G*CTAGC*T*ACAAC*G*ATG/i2OMeC//i2OMeA//i2OMeC//i2OMeG//i2OMeC//i2OMeC//3idT/ |
| 69Q-70C 5MOERNA-6PS 5Cy5 3idT (8 nt binding arm) | /5Cy5//i2MOErC//i2MOErT//i2MOErG//i2MOErA//i2MOErA/GCAG*G*CTAGC*T*ACAAC*G*ATGC/i2MOErA//i2MOErC//i2MOErG//i2MOErC//i2MOErC//3idT/ |
| 176V 19 nt all RNA substrate | /56FAM/rGrArCrGrGrCrArGrCrGrUrGrCrArGrCrUrCrG |
| 176V WT 7-7 idT (7 nt binding arm) | AGCTGCAGGCTAGCTACAACGAGCTGCCG/3idT/ |
| 176V 6/7FRNA-6PS 5Cy5 3idT (7 nt binding arm) [FRNA-6PS] | /5Cy5//i2FA//i2FG//i2FC//i2FU//i2FG//i2FC/dAdG*dG*dCdTdAdGdC*dT*dAdCdAdAdC*dG*dA/i2FG//i2FC//i2FU//i2FG//i2FC//i2FC//i2FG//3idT/ |
| 176V 6OMeRNA-6PS 5Cy5 3idT (7 nt binding arm) [OMeRNA-6PS] | /5Cy5//i2OMeA//i2OMeG//i2OMeC//i2OMeU//i2OMeG//i2OMeC/AG*G*CTAGC*T*ACAAC*G*AG/i2OMeC//i2OMeU//i2OMeG//i2OMeC//i2OMeC//i2OMeG//idT/ |
| 176V 6OMeRNA-6PS 5Cy5 3idT (8 nt binding arm) | /5Cy5//i2OMeG//i2OMeA//i2OMeG//i2OMeC//i2OMeU//i2OMeG/CAG*G*CTAGC*T*ACAAC*G*AGC/i2OMeU//i2OMeG//i2OMeC//i2OMeC//i2OMeG//i2OMeU//3idT/ |
| 176V 5MOERNA-6PS 5Cy5 3idT (8 nt binding arm) [MOERNA-6PS] | /5Cy5//i2MOErG//i2MOErA//i2MOErG//i2MOErC//i2MOErT/GCAG*G*CTAGC*T*ACAAC*G*AGCT/i2MOErG//i2MOErC//i2MOErC//i2MOErG//i2MOErT//3idT/ |
| 176V 18 nt biotinylated RNA strand | /5Biotin/rGrArCrGrGrCrArGrCrUrGrCrArGrCrUrCrG |
| Oligonucleotides for c-MYC inhibition | |
| Myc-1 FRNA-6PS | /i2FC//i2FC//i2FC//i2FA//i2FG//i2FG/AG*G*CTAGC*T*ACAAC*G*A/i2FG//i2FC//i2FC//i2FC//i2FG//i2FC//i2FA//3idT/ |
| Myc-1 OMeRNA-6PS | /i2OMeC//i2OMeC//i2OMeC//i2OMeA//i2OMeG//i2OMeG/AG*G*CTAGC*T*ACAAC*G*AG/i2OMeC//i2OMeC//i2OMeC//i2OMeG//i2OMeC//i2OMeA//3idT/ |
| Myc-1 MOERNA-6PS | /i2MOErT//i2MOErC//i2MOErC//i2MOErC//i2MOErA/GGAG*G*CTAGC*T*ACAAC*G*AGCC/i2MOErC//i2MOErG//i2MOErC//i2MOErA//i2MOErG//3idT/ |
| Myc-2 FRNA-6PS | /i2FA//i2FA//i2FG//i2FC//i2FU//i2FA/AG*G*CTAGC*T*ACAAC*G*A/i2FG//i2FU//i2FU//i2FG//i2FA//i2FG//i2FG//3idT/ |
| Myc-3 FRNA-6PS | /i2FU//i2FG//i2FG//i2FU//i2FU//i2FC/AG*G*CTAGC*T*ACAAC*G*A/i2FC//i2FA//i2FU//i2FG//i2FU//i2FC//i2FU//3idT/ |
| Myc-4 FRNA-6PS | /i2FG//i2FC//i2FA//i2FG//i2FG//i2FU/AG*G*CTAGC*T*ACAAC*G*A/i2FA//i2FA//i2FG//i2FC//i2FU//i2FG//i2FG//3idT/ |
| Myc-5 FRNA-6PS | /i2FG//i2FU//i2FG//i2FG//i2FA//i2FG/AG*G*CTAGC*T*ACAAC*G*A/i2FG//i2FU//i2FG//i2FG//i2FC//i2FA//i2FC//3idT/ |
| Myc-6 FRNA-6PS | /i2FC//i2FU//i2FC//i2FA//i2FG//i2FG/AG*G*CTAGC*T*ACAAC*G*A/i2FU//i2FC//i2FU//i2FG//i2FA//i2FC//i2FA//3idT/ |
| Myc-7 FRNA-6PS | /i2FA//i2FG//i2FG//i2FA//i2FU//i2FA/AG*G*CTAGC*T*ACAAC*G*A/i2FU//i2FA//i2FC//i2FC//i2FU//i2FU//i2FG//3idT/ |
| Myc-8 FRNA-6PS | /i2FU//i2FC//i2FC//i2FA//i2FC//i2FU/AG*G*CTAGC*T*ACAAC*G*A/i2FC//i2FC//i2FG//i2FA//i2FA//i2FA//i2FA//3idT/ |

Phosphorothioate (PS) linkages in the backbone structure are denoted by “*”; RNA residues are denoted by “r”; “3idT” denotes 3’ inverted dT; “i2FN” denotes 2'-Fluoro ribonucleotides; “i2OMeN” denotes 2'-O-methyl ribonucleotides; “i2MOErN” denotes 2’-O-methoxyethyl ribonucleotides.

**Table S2.** Table of primers used for qPCR

| Primer ID | Sequence (5' to 3') | Target |
| --- | --- | --- |
| GAPDH Q-For | ACCATCTTCCAGGAGCGAGATCCCTC | GAPDH |
| GAPDH Q-Rev | TGCAGGAGGCATTGCTGATGATCTTGA | GAPDH |
| 69-70 Q-For | CCCTGACCTACGGCGTGCAGTGCTTC | eGFP |
| 69-70 Q-Rev | CGCTCCTGGACGTAGCCTTCGGGC | eGFP |
| 176V Q-For | CAACATCGAGGACGGCAGCGTGCAGC | eGFP |
| 176V Q-Rev | GCTCAGGTAGTGGTTGTCGGGCAG | eGFP |
| Myc-1 For | AGTAATTCCAGCGAGAGGCAGAGGGA | Myc |
| Myc-1 Rev | GTTGCGGACCGCTGGCTGGGGGAT | Myc |
| Myc-2 For | AAGGCTCTCCTTGCAGCTGCTTAGAC | Myc |
| Myc-2 Rev | GGTAGAAGTTCTCCTCCTCGTCGC | Myc |
| Myc-3 For | GGGGAGACAACGACGGCGGTGG | Myc |
| Myc-3 Rev | GAGAAGCCGCTCCACATACAGTCCT | Myc |
| Myc-4 For | GCTGGCCTCCTACCAGGCTGCG | Myc |
| Myc-4 Rev | CGAGCTGCTGTCGTTGAGAGGGTAGG | Myc |
| Myc-5 For | GGCAAAAGGTCAGAGTCTGGATCACC | Myc |
| Myc-5 Rev | CACTGTCCAACTTGACCCTCTTGGCAG | Myc |
| Myc-6 For | ACATCAGCACAACTACGCAGCGCCTC | Myc |
| Myc-6 Rev | GTTGTGTGTTCGCCTCTTGACATTCTCC | Myc |
| Myc-7 For | GAGGAGGAACGAGCTAAAACGGAGC | Myc |
| Myc-7 Rev | CCGCAACAAGTCCTCTTCAGAAATGAGC | Myc |
| Myc-8 For | TGGGGACACTTCCCCGCCGCTGC | Myc |
| Myc-8 Rev | ACCGAGTCGTAGTCGAGGTCATAGTTCC | Myc |

**Table S3.** Table of oligos and primers used for 5’ RACE PCR

| Oligo name | Sequence (5' to 3') |
| --- | --- |
| Template switching oligo  (TSO) | GCTAATCATTGCAAGCAGTGGTATCAACGCAGAGTACATrGrGrG |
| PCR1 primers | |
| TS1 | AAGCAGTGGTATCAACGCAGAGTAC |
| eGFP-R-RACE-1 | TTACTTGTACAGCTCGTCCATGCCGAG |
| Myc-1 RACE 1 OUTER | GGCGGCCGAGAAGCCGCTCCACAT |
| PCR2 primers | |
| TS2 | AAGCAGTGGTATCAACGC |
| 176V Q-Rev | GCTCAGGTAGTGGTTGTCGGGCAG |
| Myc-1 RACE 2 INNER | GTTGCGGACCGCTGGCTGGGGGAT |

RNA residues are denoted by “r”
